# Hydrogen Atom Transfer-Triggered Chemical Recycling of Commercial Poly(methyl methacrylate) Utilizing Continuous Monomer Removal

**DOI:** 10.1021/acsmacrolett.6c00454

**Published:** 2026-09-29

**Authors:** Matylda Szewczyk-Łagodzińska, Ferdinando De Luca Bossa, Yanzhe Fang, Jakub Józiewicz, Halil Ibrahim Coskun, Krzysztof Matyjaszewski

**Affiliations:** † Department of Chemistry, 6612 Carnegie Mellon University, 4400 Fifth Avenue, Pittsburgh, Pennsylvania 15213, United States; ‡ Department of Molecular Physics, Faculty of Chemistry, Łódź University of Technology, Żeromskiego Street 114, Łódź 90-543, Poland

## Abstract

We report a thermal hydrogen atom transfer (HAT) depolymerization strategy for the efficient chemical recycling of unmodified commercial poly­(methyl methacrylate) (PMMA). A reaction engineering approach combining controlled radical concentration with continuous monomer removal enables efficient depolymerization under practical conditions without light, chlorinated solvents, or high dilution. A semibatch protocol employing multiple small additions of cumene hydroperoxide (CHP) and continuous removal of methyl methacrylate (MMA) suppressed radical side reactions and shifted the equilibrium toward monomer formation. At a repeating-unit concentration of 1.83 M and a total CHP loading of 3 mol %, depolymerization reached 90% with only 2% monomer consumption. The method remained effective at 3.00 M, scaled to 20 g of polymer (79% depolymerization, 4.5 mol % CHP loading), and was effective for commercial Plexiglas containing stabilizing additives. Recovered MMA was successfully repolymerized, demonstrating its circularity. These results establish radical concentration control and continuous monomer removal as the key design principles for hydroperoxide-assisted thermal HAT depolymerization.

## Graphical abstract

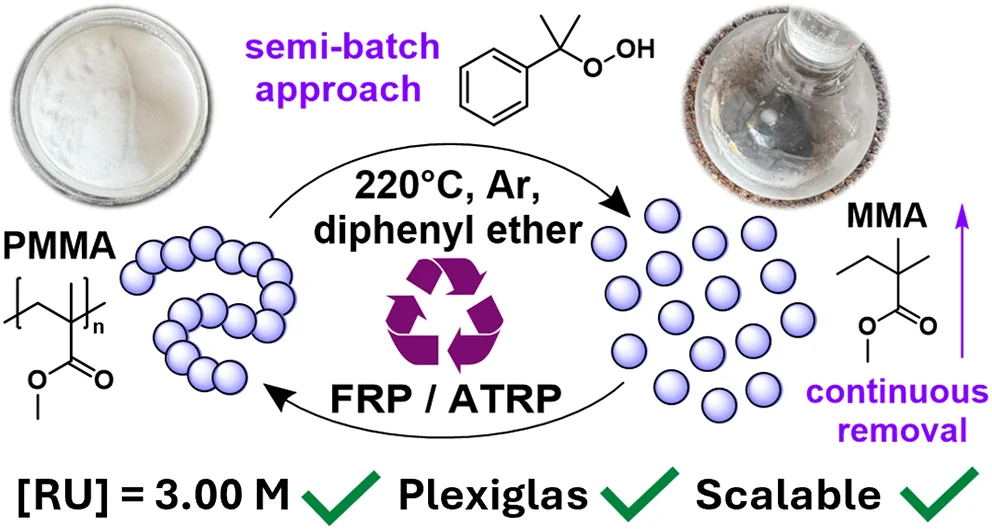

Poly­(methyl methacrylate) (PMMA) is produced on a multimillion-ton scale (∼2.6 million metric tons globally in 2025) and is widely used in construction, optical materials, and biomedical devices.^1^ Its chemical recycling, however, is challenging because of its inert carbon–carbon backbone.^2,3^ Monomer recovery currently relies on thermal pyrolysis, which proceeds through random backbone scission at temperatures typically exceeding 400 °C under oxygen-free conditions. These severe thermal requirements increase energy consumption and demand specialized equipment, motivating the search for depolymerization strategies that operate under substantially milder conditions.^4–8^

Reversible deactivation radical polymerization (RDRP) strategies lower the depolymerization temperature by exploiting well-defined dormant chain ends (alkyl halides for atom transfer radical polymerization (ATRP) or thiocarbonylthio chain transfer agents for reversible addition–fragmentation chain transfer (RAFT) polymerization) that can be reactivated to generate macroradicals.^9–11^ Above the ceiling temperature, or below the equilibrium monomer concentration, depolymerization dominates over polymerization in the presence of such radicals.^12–18^ Using this strategy, different catalysts, dilutions, temperatures,^19–28^ and external stimuli have been reported.^29–36^ Commercial PMMA, however, is generally synthesized by free-radical polymerization (FRP) and consequently lacks the tailored chain-end groups required for efficient end-initiated depolymerization. Pendant-group activation offers an alternative strategy by incorporating labile functionalities along the polymer backbone that can initiate midchain scission. Reported examples include *N*-hydroxyphthalimide and trityl-derived activated esters, and α-methylstyrene-based comonomers. Upon activation, the former two classes generate radical intermediates that can undergo decarboxylation, producing backbone radicals that fragment and initiate depolymerization. By contrast, α-methylstyrene units reduce the strength of adjacent backbone C–C bonds, thereby promoting chain scission and subsequent unzipping.^37–43^ These strategies require either a comonomer during PMMA synthesis or a postpolymerization modification. Consequently, neither RDRP nor pendant-group activation applies directly to unmodified commercial PMMA.

Photochemical hydrogen atom transfer (HAT) has recently enabled the depolymerization of commercial polymethacrylates: light-generated radicals in situ abstract hydrogen atoms from the backbone, triggering fragmentation and depolymerization. These methods, however, require short-wavelength light, very dilute solutions, and, often, chlorinated compounds/solvents.^44–48^

We recently reported a thermally driven HAT depolymerization system that addressed these limitations (Scheme 1).^49^ Simple hydroperoxides generated peroxyl and alkoxyl radicals that abstracted H atoms from the PMMA backbone without light, chlorinated solvents, or extreme dilution. At 220 °C with 10 mol % *tert*-butyl hydroperoxide (TBHP) relative to the repeat units, this system achieved up to 70% depolymerization of PMMA solution in diphenyl ether (DPE, 8 wt % of polymer). However, the alkoxyl radicals formed upon decomposition of the hydroperoxide were further degraded by β-scission to methyl radicals, which could not efficiently abstract H atoms from PMMA. These methyl radicals instead participated in termination and consumed about 15% of the monomer formed, generating oligomers and limiting the overall efficiency of the process.

**Scheme 1:**
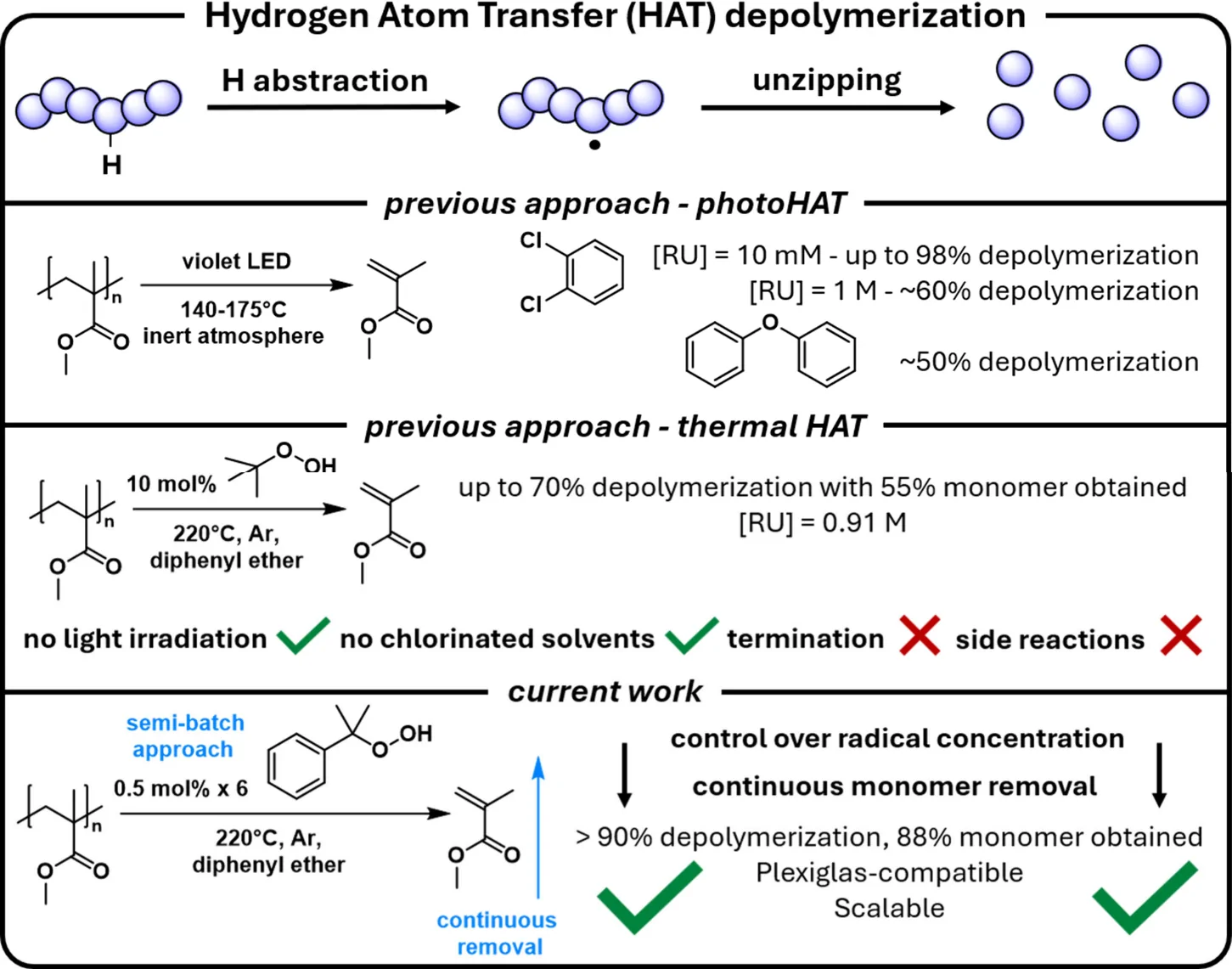
HAT Depolymerization Strategies for Commercial PMMA^a^

a Previous approach – photoHAT – reaction mixture irradiation utilizing solvent as the HAT agent; previous approach – thermal HAT - elevated temperature is used for HAT agent activation, performed in sealed vials with high radical concentration; current work – semi-batch strategy thermal HAT with continuous monomer removal.

Side reactions can be suppressed if peroxide is delivered utilizing a programmed dosing strategy, as recently reported.^50^ PMMA was depolymerized at 170 °C in a closed vial at [RU] = 10 mM (0.07 wt %), using three di*tert*-butyl peroxide (DTBP) additions, each corresponding to 0.1 DTBP eq relative to [RU]_0_ (30 mol % total). This approach afforded >95% depolymerization, however, the low polymer concentration required a large solvent volume, while the high total peroxide loading could limit scalability.

We hypothesized that the principal limitations of thermal HAT depolymerization, radical side reactions and equilibrium-limited monomer formation, could be overcome through simultaneous control of radical concentration and continuous monomer removal. Lowering the radical concentration would suppress unwanted radical side reactions, while removing monomer under reduced pressure would continuously shift the depolymerization equilibrium toward MMA, allowing the reaction to proceed beyond the equilibrium limit of conventional operations. Based on this concept, we report a semibatch protocol with several additions of small amounts of hydroperoxides, combined with continuous monomer removal (Scheme 1). As an example, cumene hydroperoxide (CHP) was introduced in six 0.5 mol % portions, accounting for a total of 3 mol % relative to the repeat units in PMMA (at [RU] = 0.91 M), representing a 3.33-fold reduction compared to our previous work and a 10-fold reduction compared to the recent reports in the field.^49,50^ Under these optimized conditions, commercial PMMA underwent 90% depolymerization with minimal 2% monomer consumption. The method was further scaled up to 20 g of polymer and applied to an 8 wt % solution of commercial Plexiglas. The recovered monomer was successfully repolymerized using FRP and ATRP (*M*_n_ = 112.4 kg/mol, *Đ* = 1.97, and *M*_n_ = 11.5 kg/mol, *Đ* = 1.10, respectively). Collectively, these results establish control over radical concentration and continuous product removal as general design principles for thermal HAT depolymerization, transforming it into a practical and scalable strategy for the chemical recycling of commercial PMMA. The method uses high polymer concentration (up to 22 wt %) under operationally simple conditions, relies on a commercially available peroxide, and efficiently depolymerizes Plexiglas despite the presence of inhibitors and stabilizers.

The reactions were performed in a short-path distillation setup connected to the vacuum pump with a pressure control system (Scheme S1, Figure S1). The pressure was adjusted to maintain reflux, while the thermometer port of the distillation head was fitted with a septum for reagent addition and reaction monitoring by an aliquot withdrawal.

Reaction progress was monitored by two complementary ^1^H NMR methods and independently verified by GPC (see Supporting Information, page 9, “Reaction Monitoring”, Figures S2 and S4–S15). The NMR analyses quantified polymer degradation and product composition, while GPC provided an independent measure of polymer degradation. The agreement between the three methods confirmed the reliability of the analysis.

The initial investigation focused on peroxide screening. Four peroxides, TBHP, DTBP, CHP, and dicumyl peroxide (DCP), were selected based on their thermal stability and boiling point. The decomposition rate determined radical concentration: rapid decomposition led to high radical concentration and promoted side reactions, while slow decomposition limited the reaction rate. Boiling point is a critical parameter under reduced pressure, as volatile peroxides can be distilled off and lost before reacting (Table S1). Screening was performed under previously optimized conditions: 10 mol % peroxide vs MMA units ([RU]), 220 °C, and 0.91 M [RU] ([MMA]_eq_ at 220 °C = 1.68 M), with three successive additions of 10 mol % at 1 h intervals (Figure 1).

**Figure 1:**
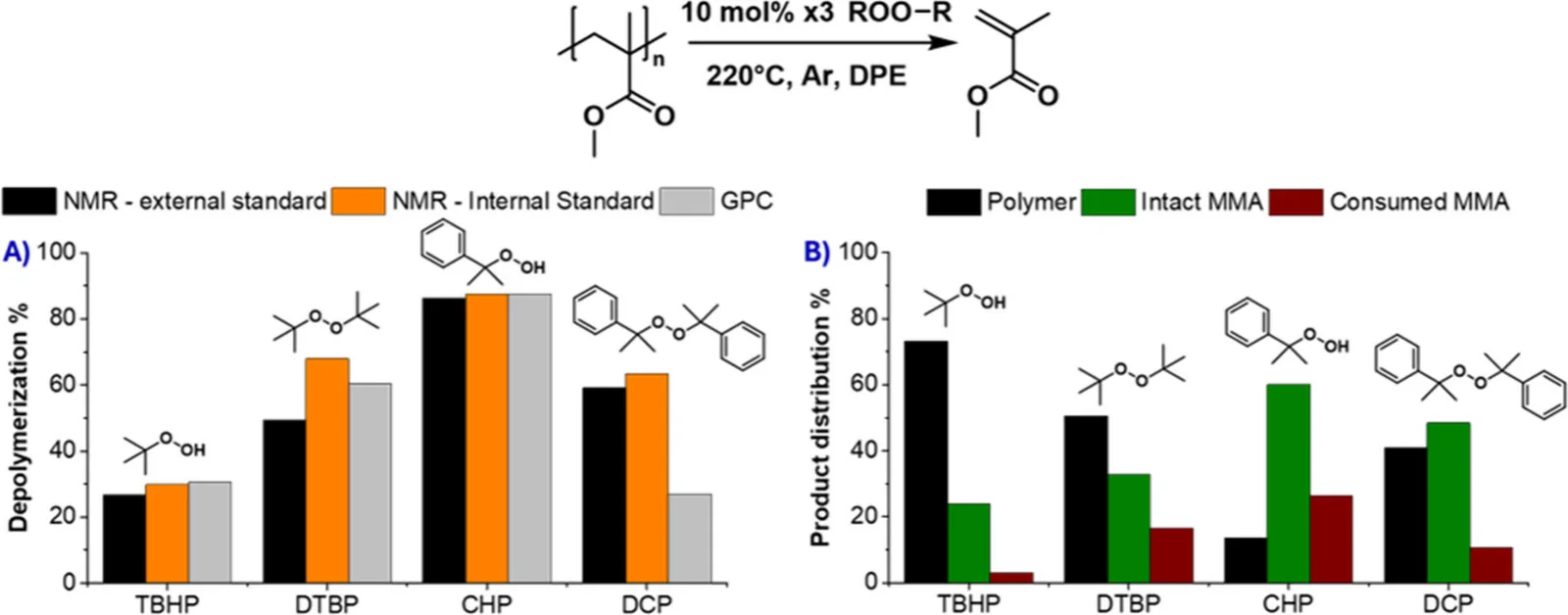
Results of the reagent screening reactions: (A) comparison of final (3 h) depolymerization results obtained through ^1^H NMR with external standard, internal standard, and GPC; (B) product distribution%, calculated using ^1^H NMR with external standard (see Supporting Information, page 9, “Reaction Monitoring”). These results are for the PMMA depolymerization at 220 °C, with three 10 mol % reagent additions respective to [RU] at *t* = 0, 1, and 2 h. [RU] = 0.91 M; 12.5 mL total reaction volume; 30 min deoxygenation prior to heating.

CHP proved to be the most promising peroxide. It gave ∼86% total polymer degradation. However, only 60% was intact monomer, while 26% was detected as consumed monomer. TBHP was expected to perform better, having a longer half-life (∼6 min) than CHP (∼3 min) at 220 °C. However, it performed considerably worse. This can be attributed to its low boiling point (96 °C), since it was partially distilled off from the reaction before it reacted, as evidenced by its presence in the ^1^H NMR spectrum of the distilled product (Figure S3). DCP and DTBP performed poorly because of their lower thermal stability; their half-lifes at 220 °C are ∼2.5 s and ∼8 s, respectively.^49,51^

Next, CHP loading was optimized to minimize monomer consumption while maintaining efficient depolymerization (Table 1). Reactions were performed using the semibatch protocol described above and continued until depolymerization reached a plateau (Figure S8). High CHP loading, 10 mol % batches, resulted in faster monomer consumption than removal. At 5 mol % CHP per addition, 92% depolymerization was achieved with only 10% consumed monomer, compared to 26% consumption at 10 mol % CHP batches (Table 1, entries 1 and 2). This validates the semibatch approach. Lower radical concentration is advantageous not only because it limits monomer consumption but also termination, leading to higher overall depolymerization %.

**Table 1:** Results of the CHP Batch-Size Optimization for the PMMA Depolymerization at 220 °C, with Semibatch CHP Additions Each Hour^a^

					depolymerization (%)			product distribution^e^ (%)		
entry	batch^b^ (mol %)	No. of batches	total mol %	batch form	NMR e.s.^c^	NMR i.s.^d^	GPC	polymer	monomer	consumed monomer
1	10	3	30	CHP	86	87	88	14	60	26
2	5	4	20	CHP	92	92	96	8	82	10
3	2.5	4	10	CHP in cumene	87	88	85	13	86	1
4	2.5	5	12.5	CHP in DPE	93	94	93	7	88	5
5	1	5	5	CHP in cumene	72	74	64	28	72	0
6	1	6	6	CHP in DPE	89	89	88	11	88	1
7	0.5	7	3.5	CHP in DPE	87	88	86	13	87	0
8	0.1	5	0.5	CHP in DPE	61	56	49	39	61	0
9	0	0	0		0	0	0	100	0	0

a The reactions were stopped when depolymerization % plateaued. [RU] = 0.91 M; 12.5 mL total reaction volume; 30 min deoxygenation prior to heating.

b Relative to that of [RU].

c NMR e.s. stands for ^1^H NMR results obtained with an external standard.

d NMR i.s. stands for ^1^H NMR results obtained with an internal standard.

e Calculated using ^1^H NMR with an external standard (see Supporting Information, page 9, “Reaction Monitoring”).

For reactions with lower CHP batches, stock solutions were prepared either in cumene or DPE. The latter consistently afforded higher depolymerization than cumene at identical CHP loadings, reaching 93% and 89% depolymerization with 2.5 mol % and 1 mol %, per addition, respectively. In comparison, in cumene, the reactions plateaued at 87% and 72% depolymerization (Table 1, entries 3–6). The superior performance of DPE is attributed to both physical and chemical factors. Under reduced pressure, the lower boiling point of cumene (152 °C) facilitated removal of CHP before productive decomposition, whereas the higher boiling point of DPE (259 °C) prolonged its residence time in the reaction mixture. Moreover, the benzylic C–H bond of cumene (∼80 kcal/mol) is weaker than the tertiary C–H bonds of PMMA (∼100 kcal/mol), making cumene a preferred hydrogen atom donor and therefore a competitive site for the radicals responsible for polymer activation.

Further reducing CHP loading to 0.5 mol % per addition resulted in 87% depolymerization after seven additions with no detectable monomer consumption. Decreasing the additive amount further, to 0.1 mol %, proved insufficient for depolymerization, which plateaued at 61% (Table 1, entries 7 and 8).

To prove that depolymerization was initiated by HAT and not purely thermally, a control reaction without CHP was performed (Table 1, entry 9). The reaction mixture was stirred for 5 h. Both ^1^H NMR and GPC showed no depletion of polymer signals or distributions after the reaction. A minuscule amount of distillate was collected, and the *M*_n_ change was minimal (from 181 to 170 kg/mol). These results support the claim that HAT is essential for the depolymerization to proceed.

Overall, when working at [RU] = 0.91 M, the total hydroperoxide loading was reduced from 10 mol % in a single batch to 3.5 mol % across multiple batches. The depolymerization was improved from 70% (with 15% of consumed monomer) to 87%, with no detectable monomer consumption. Using the hydroperoxide in small portions, rather than as a single-batch charge, also improved the safety of the method.

Continuous monomer removal should allow for performing reactions above the equilibrium monomer concentration, which for MMA at 220 °C is [MMA]_eq_ = 1.68 M. Therefore, additional reactions were carried out with [RU] = 1.83 and 3.00 M, with six and five additions of 0.5 mol % CHP batches, respectively (3 mol % and 2.5 mol % total) (Figure 2). The reaction with [RU] = 1.83 M gave comparable results to the original conditions ([RU] = 0.91 M) - 90% depolymerization with 2% monomer consumption. Further increase of [RU] gave the same depolymerization% but with slightly increased monomer consumption of 4%. This can be attributed to increased viscosity of the reaction mixture, with the monomer becoming more difficult to distill from the solution. These results show that the thermodynamic limitation of [MMA]_eq_ can be surpassed under continuous monomer removal.

**Figure 2:**
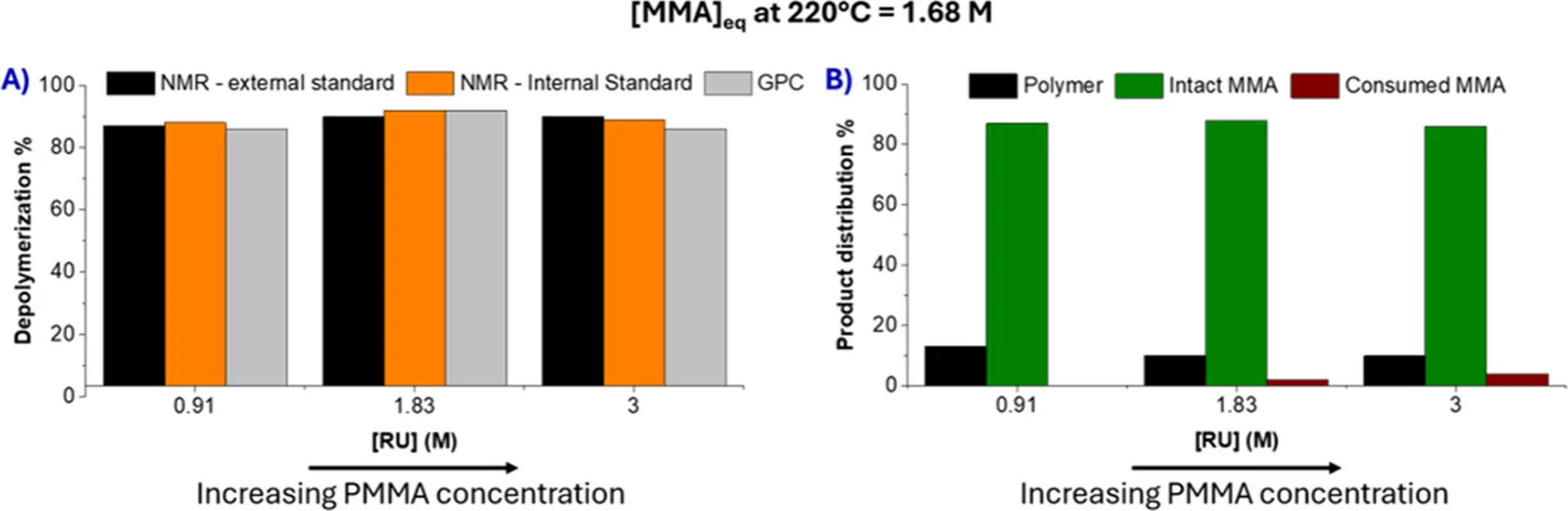
Results of the varied [RU] concentration reactions: (A) comparison of final depolymerization results obtained through ^1^H NMR with external standard, internal standard, and GPC; (B) product distribution %, calculated using ^1^H NMR with external standard (see Supporting Information, page 9, “Reaction Monitoring”). These results are for the PMMA depolymerization at 220 °C, with 0.5 mol % CHP semibatch additions respective to [RU] each consecutive hour. 12.5 mL total reaction volume; 30 min deoxygenation prior to heating. Reaction with [RU] = 0.91 M was stopped after 7 h, [RU] = 1.83 M after 6 h, and [RU] = 3.00 M after 5 h.

To further explore the system, Plexiglas depolymerization was attempted by using the optimized reaction conditions (Figure 3). The higher molecular weight of Plexiglas (*M*_n_, Plexiglas = 590 kg/mol vs *M*_n_, PMMA = 180 kg/mol) made the reaction mixture significantly more viscous, impeding the monomer removal. However, the final depolymerization % using 6 × 0.5 mol % CHP batches was 81%, slightly lower than 90% achieved with commercial PMMA with the same 2% monomer consumption. Monomer formation was followed by a significant *M*_n_ reduction (95%), suggesting that depolymerization proceeds through random midchain scission initiated by the HAT agent. This shows the versatility of our method since Plexiglas contains stabilizing comonomers and inhibitors.

**Figure 3:**
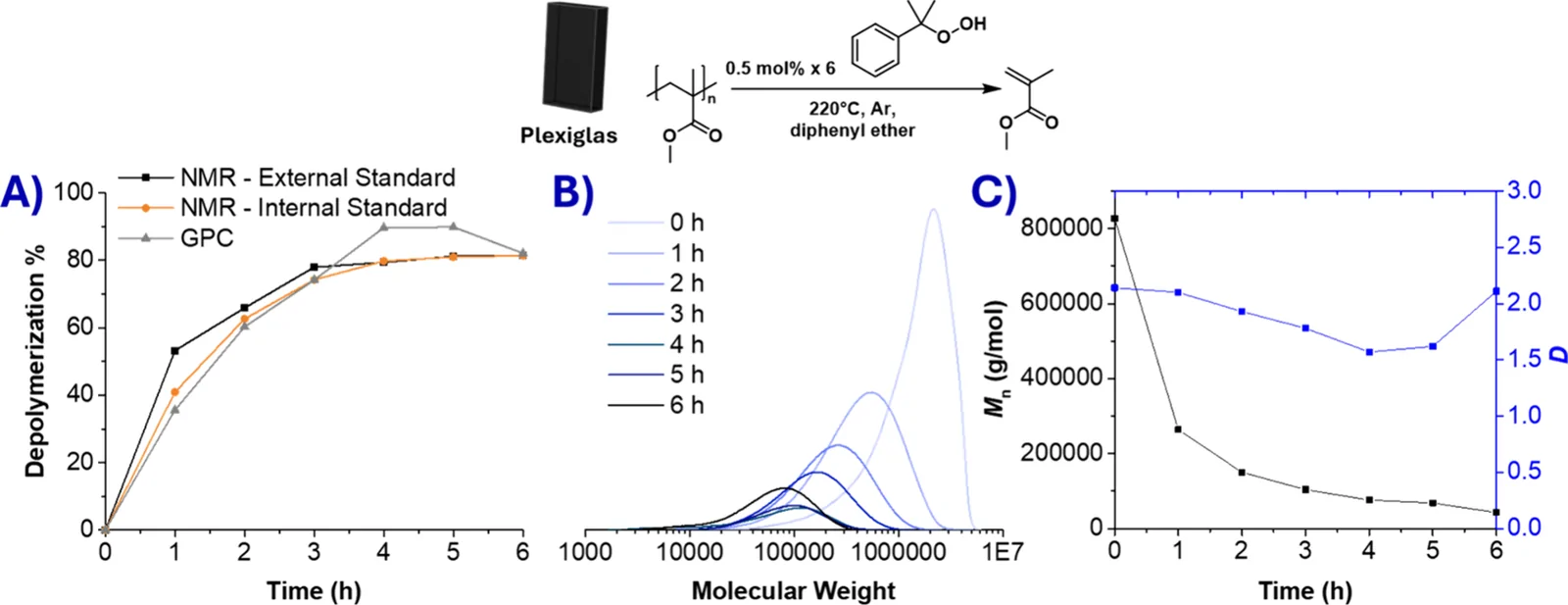
Results of the Plexiglas depolymerization reaction: (A) comparison of results obtained through ^1^H NMR with external standard, internal standard, and GPC (see Supporting Information, page 9, “Reaction Monitoring”); (B) GPC traces showing depletion of area under the curve as reaction progresses; and C) *M*_n_ and *Đ* changes in time. These results are for the Plexiglas depolymerization at 220 °C, with six 0.5 mol % CHP additions respective to [RU] after each consecutive hour. [RU] = 0.91 M; 12.5 mL total reaction volume; 30 min deoxygenation prior to heating.

The next test was the scaled-up reaction, followed by polymerization of recovered MMA. A total of 20 g of PMMA was depolymerized at [RU] = 1.83 M with nine 0.5 mol % CHP additions (Figures 4 and S16). Final depolymerization reached 79% with 1.5% consumed MMA. Considering the depolymerization extent, the final yield of MMA reached 88%. If the depolymerization extent was not considered, the yield was 68% (13.9 g of purified MMA was obtained from 20.4 g of PMMA). MMA was purified from DPE by distillation (Figure S19). The purified monomer was polymerized using ATRP and FRP, with ATRP reaching ∼90% conversion (*M*_n_ = 11.5 kg/mol, *Đ* = 1.10), while FRP gave ∼80% conversion (*M*_n_ = 112.4 kg/mol, *Đ* = 1.97). Those results confirm the validity of thermal HAT depolymerization as a potential chemical recycling method for PMMA.

**Figure 4:**
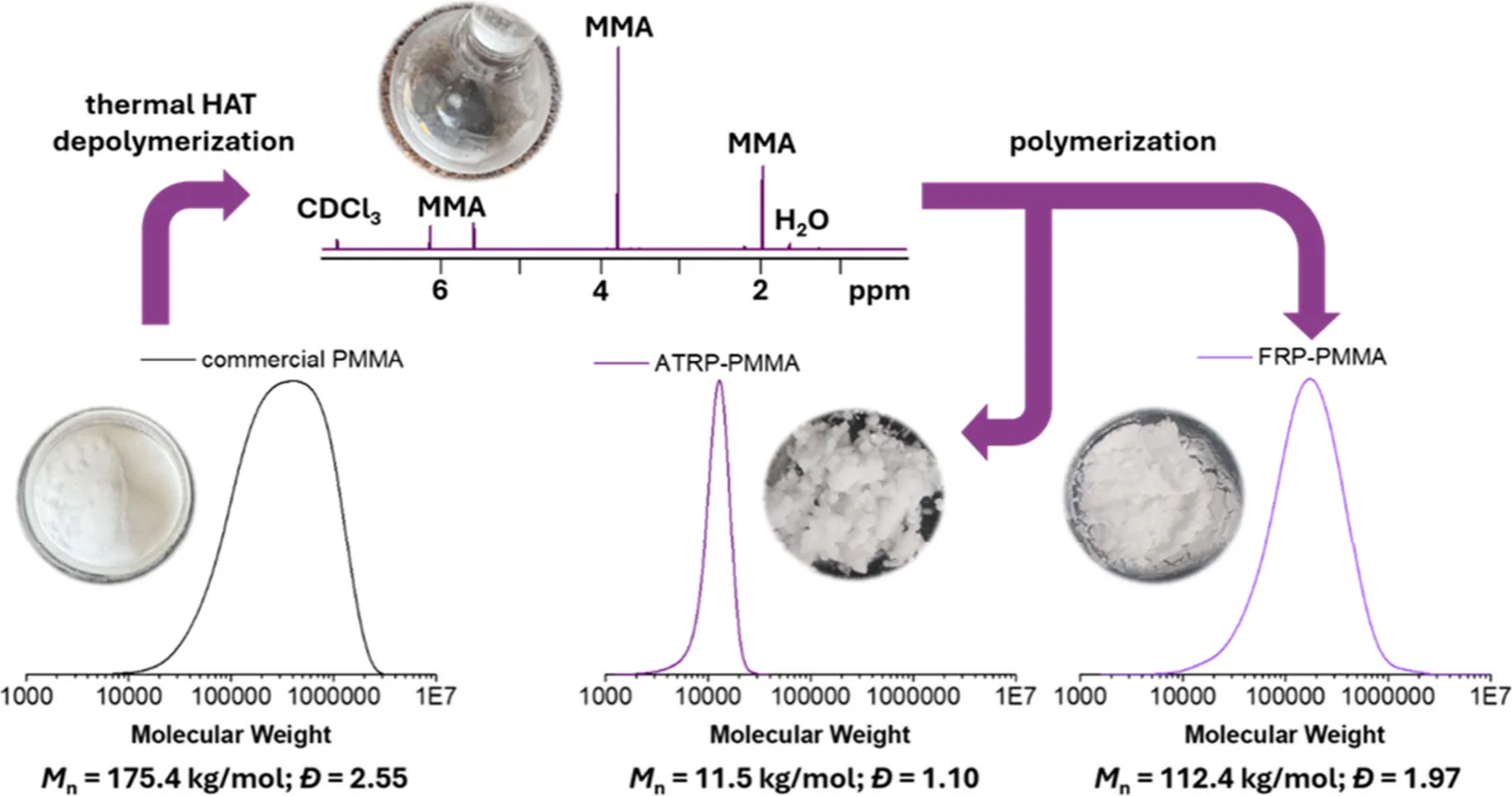
Results of the large-scale depolymerization and subsequent polymerization reactions, including the GPC chromatogram for commercial PMMA, ^1^H NMR spectrum of the purified MMA, and GPC chromatograms of the PMMA repolymerized by ATRP and FRP. These results are for the PMMA depolymerization at 220 °C, with nine 0.5 mol % CHP additions respective to [RU] after each consecutive hour. [RU] = 1.83 M; 100 mL total reaction volume; 30 min deoxygenation prior to heating. ARGET ATRP: 70 °C, 2 mL total reaction volume with 47% v/v anisole. Ethyl-α-chlorophenyl acetate:MMA:CuCl_2_:PMDETA = 1:200:0.1:0.175. One drop of tin­(II) 2-ethylhexanoate was added as a reducing agent. FRP: 70 °C, 2 mL total reaction volume with 48% v/v anisole. AIBN:MMA = 1:200. Both polymerizations were preceded by 20 min of degassing.

We developed a reaction engineering strategy for thermal HAT depolymerization that enables the practical chemical recycling of unmodified commercial PMMA. By integrating process control over radical concentration with continuous monomer removal, we overcame the key limitations of previous thermal HAT depolymerization approaches, namely, radical side reactions, equilibrium-limited monomer formation, high peroxide load, and/or low PMMA concentration. This reduced the hydroperoxide loading to 3 mol % relative to the repeat units and enabled up to 90% depolymerization with minimal monomer consumption. The process operates at high polymer concentrations, employs a commercially available peroxide, is scalable, and remains effective for Plexiglas. The recovered MMA was successfully repolymerized. More broadly, this work establishes radical concentration control and continuous product removal as general design principles for the efficient thermal depolymerization of PMMA.

## Supplementary Material



## Notes

Experimental details, ^1^H NMR spectra, and reaction monitoring data (NMR and GPC) (PDF)

## Abbreviations

HAT: hydrogen atom transfer
PMMA: poly­(methyl methacrylate)
CHP: cumene hydroperoxide
MMA: methyl methacrylate
RU: repeating unit
RDRP: reversible deactivation radical polymerization
ATRP: atom transfer radical polymerization
RAFT: reversible addition–fragmentation chain transfer
FRP: free-radical polymerization
TBHP: *tert*-butyl hydroperoxide
DPE: diphenyl ether
DTBP: di*tert*-butyl peroxide
DCP: dicumyl peroxide
GPC: gel permeation chromatography
NMR: nuclear magnetic resonance
