# Supplementary material for: Hydrogen Atom Transfer-Triggered Chemical Recycling of Commercial Poly(methyl methacrylate) Utilizing Continuous Monomer Removal
Source: ACS Macro Lett. 2026 Sep 29;15(10):1587–93. doi: 10.1021/acsmacrolett.6c00454 (PMC13689923; doi:10.1021/acsmacrolett.6c00454)
Supplement: Supplementary file 1 [file mz6c00454_si_001.pdf]

Supporting Information

Hydrogen Atom Transfer-Triggered Chemical Recycling of Commercial Poly(methyl methacrylate) Utilizing Continuous Monomer Removal

Matylda Szewczyk-Łagodzińska,[a] Ferdinando De Luca Bossa,[a] Yanzhe Fang,[a] Jakub Józiewicz,[a], [b] Halil Ibrahim Coskun,[a] and Krzysztof Matyjaszewski*[a]

[a] Department of Chemistry, Carnegie Mellon University, 4400 Fifth Avenue, Pittsburgh, PA, 15213 USA

[b] Department of Molecular Physics, Faculty of Chemistry, Łódź University of Technology, Żeromskiego Street 114, Łódź, 90-543 Poland

* E-mail: km3b@andrew.cmu.edu

Table of Contents

1.1. Materials ... 6

1.2. Instrumentation ... 6

1.3. Depolymerization ... 7

1.4. Polymerization ... 11

Table of Figures

Figure S1 Real-life depolymerization set-up with continuous monomer removal and semi-batch peroxide additions. ... 8

Figure S2 Example of ^{1}H NMR spectra used to monitor the depolymerization progress. The PMMA 3.6 ppm signal area was compared to the ethylene carbonate 4.5 ppm signal area (external standard) and to the diphenyl ether 7.1 ppm signal area (internal standard). ... 9

Figure S3 1H NMR spectrum of liquid collected in the receiving flask for the reaction with TBHP as a HAT agent. Presence of a high-intensity sharp singlet at 1.3 ppm confirms that TBHP was distilled from the reaction mixture. ... 13

Figure S4 Results of the reagent screening depolymerization reactions - comparison of results obtained through ^{1}H NMR with external standard, internal standard, and GPC for A) TBHP; B) DTBP; C) CHP; and D) DCP. These results are for the PMMA depolymerizations at 220 °C, with three 10 mol% reagent additions respective to [RU] after each consecutive hour. [RU] = 0.91 M; 12.5 ml total reaction volume; 30 min deoxygenation prior to heating. ... 14

Figure S5 Results of the reagent screening depolymerization reactions - conversion of PMMA into MMA and consumed monomer, calculated using ^{1}H NMR with an external standard, for A) TBHP; B) DTBP; C) CHP; and D) DCP. These results are for the PMMA depolymerizations at 220 °C, with three 10 mol% reagent additions respective to [RU] after each consecutive hour. [RU] = 0.91 M; 12.5 ml total reaction volume; 30 min deoxygenation prior to heating. ... 15

Figure S6 Results of the reagent screening depolymerization reactions - GPC traces showing depletion of area under the curve as reaction progresses for A) TBHP; B) DTBP; C) CHP; and D) DCP. These results are for the PMMA depolymerizations at 220 °C, with three 10 mol% reagent additions respective to [RU] after each consecutive hour. [RU] = 0.91 M; 12.5 ml total reaction volume; 30 min deoxygenation prior to heating. 16

Figure S7 Results of the reagent screening depolymerization reactions - M_n and $Đ$ changes as reaction progresses for A) TBHP; B) DTBP; C) CHP; and D) DCP. These results are for the PMMA depolymerizations at 220 °C, with three 10 mol% reagent additions respective to [RU] after each consecutive hour. [RU] = 0.91 M; 12.5 ml total reaction volume; 30 min deoxygenation prior to heating. 17

Figure S8 Results of the CHP batch-size optimization for the PMMA depolymerization at 220 °C, with semi-batch CHP additions each hour - comparison of results obtained through ^{1}H NMR with external standard, internal standard, and GPC for batch sizes relative to [RU] A) 10 mol%; B) 5 mol%; C) 2.5 mol% (CHP in cumene); D) 2.5 mol% (CHP in DPE); E) 1 mol% (CHP in cumene); F) 1 mol% (CHP in DPE); G) 0.5 mol% (CHP in DPE); and H) 0.1 mol% (CHP in DPE). The reactions were stopped when depolymerization % plateaued. [RU] = 0.91 M; 12.5 ml total reaction volume; 30 min deoxygenation prior to heating. 18

Figure S9 Results of the CHP batch-size optimization for the PMMA depolymerization at 220 °C, with semi-batch CHP additions each hour - conversion of PMMA into MMA and consumed monomer, calculated using ^{1}H NMR with an external standard, for batch sizes relative to [RU] A) 10 mol%; B) 5 mol%; C) 2.5 mol% (CHP in cumene); D) 2.5 mol% (CHP in DPE); E) 1 mol% (CHP in cumene); F) 1 mol% (CHP in DPE); G) 0.5 mol% (CHP in DPE); and H) 0.1 mol% (CHP in DPE). The reactions were stopped when depolymerization % plateaued. [RU] = 0.91 M; 12.5 ml total reaction volume; 30 min deoxygenation prior to heating. 19

Figure S10 Results of the CHP batch-size optimization for the PMMA depolymerization at 220 °C, with semi-batch CHP additions each hour - GPC traces showing depletion of area under the curve as reaction progresses for batch sizes relative to [RU] A) 10 mol%; B) 5 mol%; C) 2.5 mol% (CHP in cumene); D) 2.5 mol% (CHP in DPE); E) 1 mol% (CHP in cumene); F) 1 mol% (CHP in DPE); G) 0.5 mol% (CHP in DPE); and H) 0.1 mol% (CHP in DPE). The reactions were stopped when depolymerization % plateaued. [RU] = 0.91 M; 12.5 ml total reaction volume; 30 min deoxygenation prior to heating. 20

Figure S11 Results of the CHP batch-size optimization for the PMMA depolymerization at 220 °C, with semi-batch CHP additions each hour - M_n and *Đ* changes as reaction progresses for batch sizes relative to [RU] A) 10 mol%; B) 5 mol%; C) 2.5 mol% (CHP in cumene); D) 2.5 mol% (CHP in DPE); E) 1 mol% (CHP in cumene); F) 1 mol% (CHP in DPE); G) 0.5 mol% (CHP in DPE); and H) 0.1 mol% (CHP in DPE). The reactions were stopped when depolymerization % plateaued. [RU] = 0.91 M; 12.5 ml total reaction volume; 30 min deoxygenation prior to heating. 21

Figure S12 Results of the varied [RU] concentration depolymerization reactions - comparison of results obtained through ^{1}H NMR with external standard, internal standard, and GPC for A) [RU] = 0.91 M; B) [RU] = 1.83 M; and C) [RU] = 3.00 M. These results are for the PMMA depolymerization at 220 °C, with 0.5 mol% CHP semi-batch additions respective to [RU] each consecutive hour. 12.5 ml total reaction volume; 30 min deoxygenation prior to heating. Reaction with [RU] = 0.91 M was stopped after 7 h, [RU] = 1.83 M after 6 h, and [RU] = 3.00 M after 5 h. 22

Figure S13 Results of the varied [RU] concentration depolymerization reactions - conversion of PMMA into MMA and consumed monomer, calculated using ^{1}H NMR with an external standard for A) [RU] = 0.91 M; B) [RU] = 1.83 M; and C) [RU] = 3.00 M. These results are for the PMMA depolymerization at 220 °C, with 0.5 mol% CHP semi-batch additions respective to [RU] each consecutive hour. 12.5 ml total reaction volume; 30 min deoxygenation prior to heating. Reaction with [RU] = 0.91 M was stopped after 7 h, [RU] = 1.83 M after 6 h, and [RU] = 3.00 M after 5 h. 23

Figure S14 Results of the varied [RU] concentration depolymerization reactions - GPC traces showing depletion of area under the curve as reaction progresses for A) [RU] = 0.91 M; B) [RU] = 1.83 M; and C) [RU] = 3.00 M. These results are for the PMMA depolymerization at 220 °C, with 0.5 mol% CHP semi-batch additions respective to [RU] each consecutive hour. 12.5 ml total reaction volume; 30 min deoxygenation prior to heating. Reaction with [RU] = 0.91 M was stopped after 7 h, [RU] = 1.83 M after 6 h, and [RU] = 3.00 M after 5 h. 24

Figure S15 Results of the varied [RU] concentration depolymerization reactions - M_n and *Đ* changes as reaction progresses for A) [RU] = 0.91 M; B) [RU] = 1.83 M; and C) [RU] = 3.00 M. These results are for the PMMA depolymerization at 220 °C, with 0.5 mol% CHP semi-batch additions respective to [RU] each consecutive hour. 12.5 ml total reaction volume; 30 min deoxygenation prior to heating. Reaction with [RU] = 0.91 M was stopped after 7 h, [RU]

= 1.83 M after 6 h, and [RU] = 3.00 M after 5 h. 25

Figure S16 Results of the polymerization reactions. 1H NMR spectra showing conversion calculations for A) ATRP; and B) FRP. ARGET ATRP: 70 °C, 2 mL total reaction volume with 47% v/v anisole. Ethyl-**α**-chlorophenyl acetate : MMA : $CuCl_2$: PMDETA = 1 : 200 : 0.1 : 0.175. One drop of tin(II) 2-ethylhexanoate was added as a reducing agent. FRP: 70 °C, 2 mL total reaction volume with 48% v/v anisole. AIBN : MMA = 1 : 200. Both polymerizations were preceded by 20 min of degassing. 26

Figure S17 Results of the repeated depolymerization reaction with DCP. A) Comparison of results obtained through ^{1}H NMR with external standard, internal standard, and GPC. Comparison of original and repetition result from B) ^{1}H NMR with external standard; C) ^{1}H NMR with internal standard; and D) GPC. These results are for the PMMA depolymerizations at 220 °C, with three 10 mol% reagent additions respective to [RU] after each consecutive hour. [RU] = 0.91 M; 12.5 ml total reaction volume; 30 min deoxygenation prior to heating. .27

Figure S18 Results of the repeated depolymerization reaction with [RU] = 1.83 M. A) Comparison of results obtained through ^{1}H NMR with external standard, internal standard, and GPC. Comparison of original and repetition result from B) ^{1}H NMR with external standard; C) ^{1}H NMR with internal standard; and D) GPC. These results are for the PMMA depolymerizations at 220 °C, with six 0.5 mol% reagent additions respective to [RU] after each consecutive hour. [RU] = 1.83 M; 12.5 ml total reaction volume; 30 min deoxygenation prior to heating. 28

Figure S19 Comparison of crude product of depolymerization reaction with purified product. These results are for the PMMA depolymerization at 220 °C, with six 0.5 mol% reagent additions respective to [RU] after each consecutive hour. [RU] = 1.83 M; 12.5 ml total reaction volume; 30 min deoxygenation prior to heating, followed by product purification by distillation. 29

Scheme S1 The schematic set-up of depolymerization reactions with continuous monomer removal and semi-batch peroxide additions. 8

Equation S1 Ceiling temperature..12

Equation S2 Equilibrium monomer concentration..........12

Table S1 Structures, forms, boiling points, and half-lifetimes at 220 °C of peroxides examined as potential HAT agents..........12

Experimental Section

1.1. Materials

All chemicals were commercially available and used without any further purification unless otherwise specified. Diphenyl ether (DPE) was purchased from Fisher Chemical. Poly(methyl methacrylate) (PMMA) was purchased from Beantown Chemical. Colored cast acrylic (poly(methyl methacrylate), PMMA) sheets (Lesnlok, ASIN: B0CZ5HX6VT; 5 × 7 in., 1/8 in. thick, 3 mm) were purchased from Amazon and used as received. The grey sheet was used for this study (M_n = 590 kg/mol, M_p = 1,800 kg/mol, $Đ$ = 2.2). It was characterized via ^{1}H NMR and gel permeation chromatography (GPC) in THF, calibrated with PMMA standards. It had a molecular weight M_n = 175 kg/mol and $Đ$ = 2.55. t-Butyl hydroperoxide (TBHP) and cumene hydroperoxide (CHP) were purchased from Aldrich. The former was a solution 70 % w/w in water, while the latter was 80 % w/w in cumene. Copper(II) chloride ($CuCl_2$), ethylene carbonate (EC), dicumyl peroxide (DCP), di-*tert*-butyl peroxide (DTBP), N,N,N′,N″,N″-pentamethyldiethylenetriamine (PMDETA), and tin(II) 2-ethylhexanoate ($Sn(EH)_2$ were purchased from Aldrich. Azobisisobutyronitrile (AIBN) was purchased from Aldrich and recrystallized in methanol before use. Tetrahydrofuran (THF, HPLC grade) was purchased from Thermo Scientific. Anisole was purchased from Oakwood Chemicals. Chloroform-D (D, 99,8%) was purchased from Cambridge Isotope Laboratories, Inc.

1.2. Instrumentation

Nuclear Magnetic Resonance (NMR) Spectroscopy

^{1}H NMR spectra were recorded using a Bruker Advance 500 MHz NMR spectrometer. $CDCl_3$ was used as a solvent, while the residual $CHCl_3$ signal served as a reference.

Gel Permeation Chromatography (GPC)

The apparent number-average molar masses (M_n) and dispersity ($Đ$) of polymers as well as the samples from the kinetic analysis were measured relative to PMMA standards using an Agilent 1260 Infinity II isocratic pump, a column set containing 3 PSS analytical columns (GRAM Lux 102, 103, 104, 105 Å pore sizes). The eluent was THF, operated at 35 °C with a flow rate of 1 mL min^{-1}.

1.3. Depolymerization

General procedure

All reactions were carried out under an inert atmosphere. Glassware was oven-dried before use, and deoxygenation was applied by bubbling argon in the system for 30 minutes before introducing any reagent. Nylon zip ties, grease, parafilm, tape, and clamps were employed to secure the system.

A stock solution of PMMA in DPE (200 mg/ml) was prepared. This solvent has a high boiling point (259°C) and no abstractable hydrogen atoms to avoid interference with the HAT process. For a typical depolymerization, DPE and PMMA stock solution were added to the 50 mL round-bottom flask equipped with a stirring bar. The flask was connected to the short-path distillation condenser (Scheme S1, Figure S1) equipped with a septum at the top and a 25 mL round-bottom flask at the bottom. The condenser was cooled with running water and attached to a vacuum pump with a pressure control system. Between the condenser and the pump, a vacuum trap cooled with liquid nitrogen was placed. Through the septum, the system was bubbled with argon for 30 min. Then, the 50 mL round-bottom flask was fully submerged in an oil bath set to 220 °C. The 25 mL round-bottom flask was placed in a dry-ice bath such that only ~1/3 of it was covered in ice. The reaction mixture was left to stir at high T for 5 min. Then, the pressure on the vacuum pump was set such that DPE was boiling and condensing simultaneously. The initial sample was taken, and 1st batch of peroxide was added, marking the start of the reaction. The reactions were monitored every hour with ^{1}H NMR and GPC. The reactions were terminated once an apparent plateau was observed, based on the ^{1}H NMR analysis.

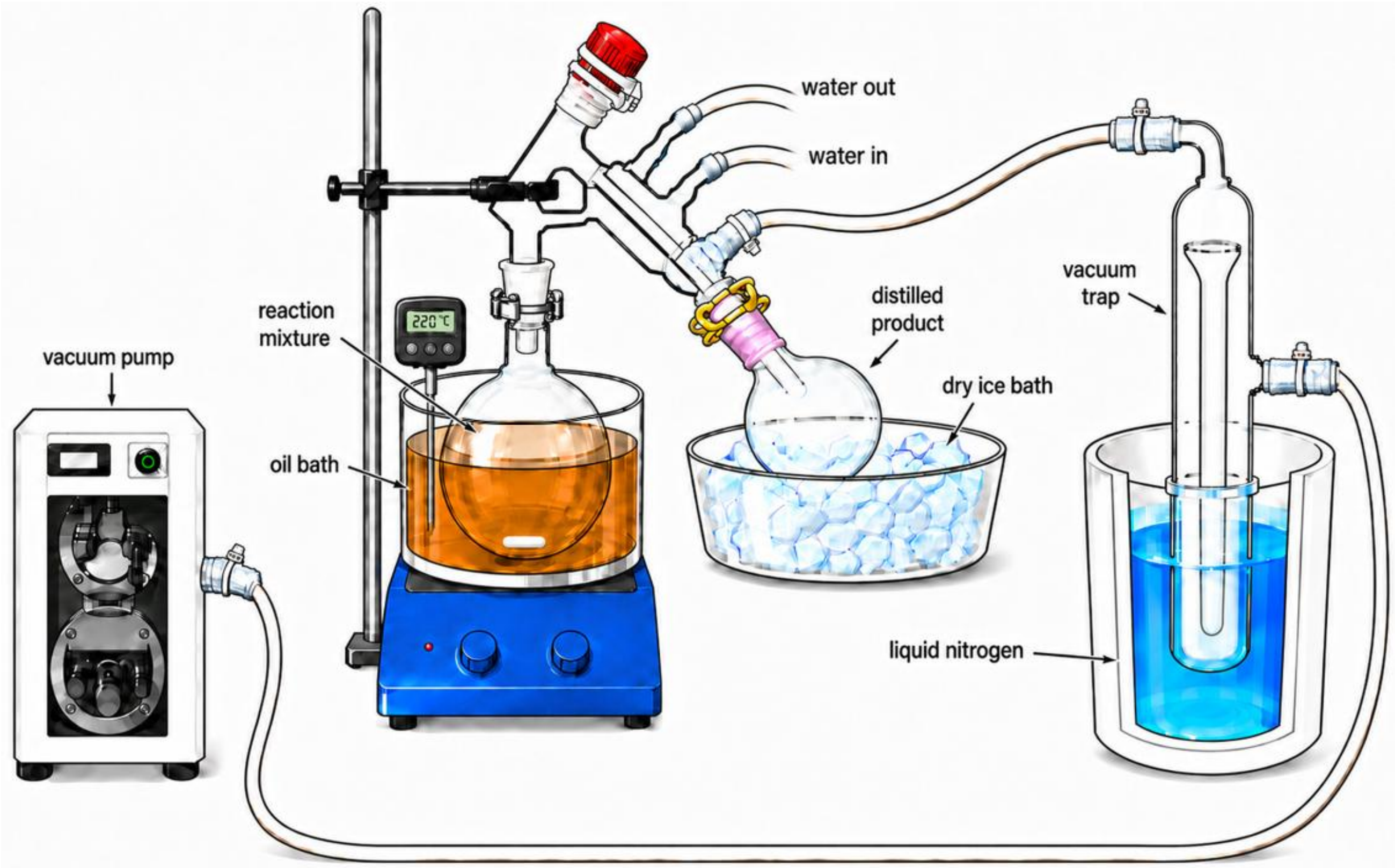

Scheme S1 The schematic set-up of depolymerization reactions with continuous monomer removal and semi-batch peroxide additions.

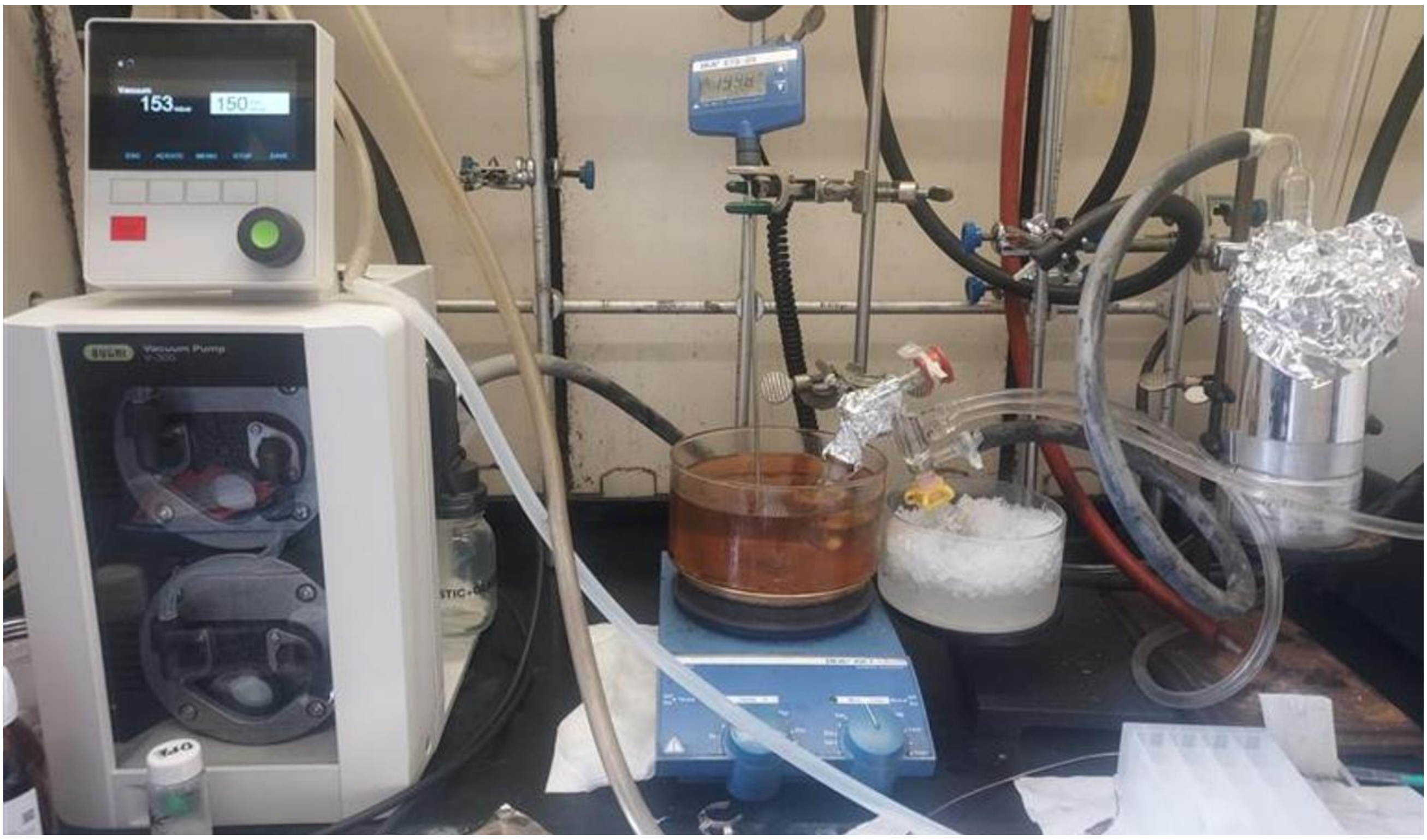

Figure S1 Real-life depolymerization set-up with continuous monomer removal and semi-batch peroxide additions.

Hazard statement: Hydroperoxides are thermally unstable, strong oxidizers that may undergo rapid exothermic decomposition at reaction conditions. Heating hydroperoxides to 220 °C under vacuum creates a risk of uncontrolled decomposition, rapid gas evolution, pressure changes, and fire/explosion. Use only appropriately rated equipment, small quantities or diluted stock solutions, adequate shielding, and suitable pressure-relief/containment measures.

Reaction Monitoring

Reaction progress was monitored by two ^{1}H NMR methods. One method used ethylene carbonate (EC) as an external standard, while the other method involved the DPE peak as an internal standard in the reaction mixture. For the external standard method, each sample was prepared using the same amount of reaction mixture (10 µL), EC (10 µL stock solution in $CDCl_3$, with 80 mg/mL concentration), and $CDCl_3$ (680 µL). Next, the PMMA 3.6 ppm and EC 4.5 ppm peaks were integrated, allowing monitoring of the decrease of the polymer peak as the reaction progressed. This method was also used to calculate the reaction products' composition. The second method utilized ^{1}H NMR spectroscopy with the diphenyl ether DPE 7.1 ppm peak as an internal standard. This peak was chosen as it was clearly distinguishable from other aromatic peaks and $CDCl_3$ (Figure S2). Moreover, GPC was used. All the samples were prepared with the same amount of reaction mixture and solvent (50 µL and 1.5 mL, respectively); hence, the areas under the chromatograms could be compared to quantitatively measure polymer degradation.

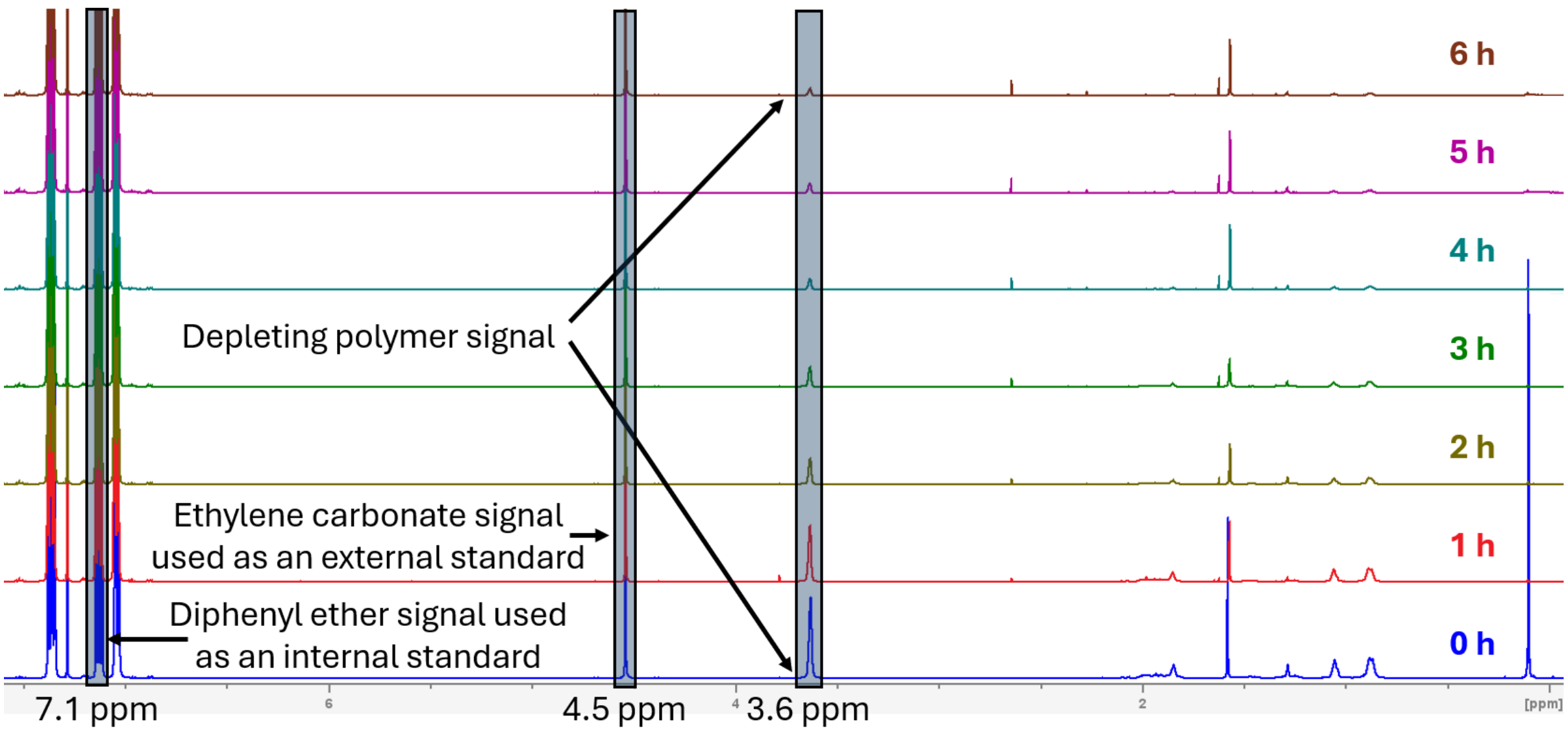

Figure S2 Example of ^{1}H NMR spectra used to monitor the depolymerization progress. The PMMA 3.6 ppm signal area was compared to the ethylene carbonate 4.5 ppm signal area (external standard) and to the diphenyl ether 7.1 ppm signal area (internal standard).

Semi-batch Additions

Peroxides were added through a septum at the top of the reaction set-up. They were added directly into the reaction mixture through a long needle. If the calculated amount of peroxide would be lower than 0.1 mL, its stock solution would be added instead. The concentrations of stock solutions were chosen such that the added amount would = 0.1 mL, the smallest amount that could be reliably added. The reactions were 12.5 mL in volume; the 0.1 mL is 0.8% of that. Introducing such a small amount of liquid did not significantly affect the reaction monitoring results.

Reagent Screening Depolymerizations

Reactions were set up in such a way that [RU] = 0.91 M and total initial reaction volume = 12.5 mL. Reagents were added in 10 mol% batches respective to [RU]. TBHP, DTBP, and CHP were added as liquids. DCP was added as a 1 mL stock solution in DPE.

CHP Batch-Size Optimization

Reactions were set up in such a way that [RU] = 0.91 M and total initial reaction volume = 12.5 mL. CHP was added as batches with x mol% relative to [RU]. For 10 and 5 mol %, CHP was added as a liquid. For 2.5 and 1 mol%, it was added either as a stock solution in cumene or DPE. For 0.1 and 0.1 mol%, it was added as a solution in DPE only.

Varied [RU] Concentration Reactions

Reactions were set up in such a way that [RU] = 0.91, 1.83, or 3.00 M, and total initial reaction volume = 12.5 mL. CHP was added in batches with 0.5 mol% relative to [RU]. The batches were 0.1 mL stock solutions of CHP in DPE. For the [RU] = 3.00 M reaction, instead of using a PMMA stock solution, PMMA was weighed directly into the round-bottom flask, and 12.5 mL of DPE were added.

Plexiglas Depolymerization Reaction

The reaction was set up in such a way that [RU] = 0.91, and the total initial reaction volume = 12.5 mL. CHP was added in batches with 0.5 mol% relative to [RU]. The batches were 0.1 mL stock solutions of CHP in DPE. Crushed black Plexiglas sheet fragments were weighed directly into the round-bottom flask, and 12.5 mL of DPE were added.

Large Scale Depolymerization

The reaction was set up in such a way that [RU] = 0.91, and with 20 g of PMMA. CHP was added in batches with 0.5 mol% relative to [RU]. The batches were 1 mL stock solutions of CHP in DPE (0.8% of reaction volume, as in small-scale reactions). PMMA was weighed directly into a 500 mL round-bottom flask, and 100 mL of DPE was added. The product was collected in a 250 mL flask. Part of the product was purified by distillation, using the same apparatus as for the depolymerization reaction (Vigreux column, ~50 ^{o}C, ~150 mbar). Temperature and pressure were adjusted during purification to promote distillation.

1.4. Polymerization

Conventional Radical Polymerization

Methyl methacrylate (1 mL, 0.94 g, 9.4 mmol) was dissolved in Anisole (0.95 mL, 48% v/v) in a 2-dram pressure release vial. AIBN (7.7 mg, 47.0 mmol, 0.005 mol% relative to the monomer) and DMF (50 µL), used as an internal standard, were then introduced. The vial was equipped with a magnetic stir bar, sealed with a septum, and purged with argon for 20 min to remove dissolved oxygen. Polymerization was performed in a preheated oil bath at 70 °C and allowed to proceed for 7 h. Monomer conversion was evaluated by comparing the integral of a vinylic proton signal with that of the internal standard before and after the reaction. The polymerization was terminated by removing the reaction vessel from the oil bath and exposing it to air. The crude polymer was isolated by precipitation into a cold methanol/water mixture (70/30) at least twice and subsequently dried under reduced pressure.

ATRP

Methyl methacrylate (1 mL, 0.94 g, 9.4 mmol) was dissolved in Anisole (0.94 mL, 47% v/v) in a 2-dram pressure release vial. Ethyl-**α**-chloro phenyl acetate (ECPA) (9.32 mg, 47.0 µmol, 8.1 µL), $CuCl_2$/PMDETA (in a ratio 1/1.75 were added as 50 µL of a stock 28.9 mg/mL in DMF, 0.1 eq to ECPA, 1.45 mg, 4.70 µmol) were then added. The vial was equipped with a magnetic stir bar, sealed with a septum, and purged with argon for 20 min to remove dissolved oxygen. One drop of $Sn(EH)_2$ was added as a reducing agent to start polymerization. The reaction was performed in a preheated oil bath at 70 °C and allowed to proceed overnight. Monomer conversion was evaluated by comparing the integral of a vinylic proton signal with that of the internal standard before and after the reaction. The polymerization was terminated by removing the reaction vessel from the oil bath and exposing it to air. The crude polymer was isolated by precipitation into a cold methanol/water mixture (70/30) at least twice and subsequently dried under reduced pressure.

1.5. Supplementary Equations, Tables, and Figures

Equation S1 Ceiling temperature

$$T_c = \frac{\Delta H}{(\Delta S^0 + R\ln[M]_0)}$$

Equation S2 Equilibrium monomer concentration

$$\ln[M]_{eq} = \frac{\Delta H}{RT} - \frac{\Delta S^0}{R}$$

To calculate T_c and $[M]_{eq}$ for PMMA, $\Delta H^0 = -59.4$ kJ mol^{-1} and $\Delta S^0 = -124.1$ J mol^{-1} K^{-1} were used.[1,2] The data for $[MMA]_{eq}$ reported in ref 1 were reanalyzed by plotting ln[MMA] versus 1/T, rather than $\ln([MMA]_{eq}/[MMA]_0)$ versus 1/T. This way H_p^0 and ΔS_p^0 for MMA were calculated.

Table S1 Structures, forms, boiling points, and half-lifetimes at 220 °C of peroxides examined as potential HAT agents.

	**TBHP**	**DTBP**	**CHP**	**DCP**
Structure	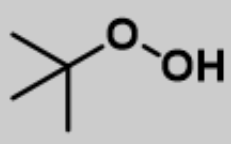	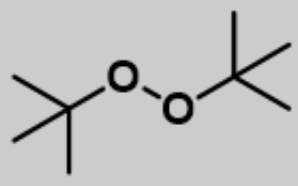	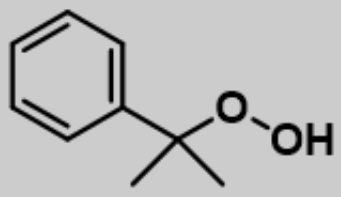	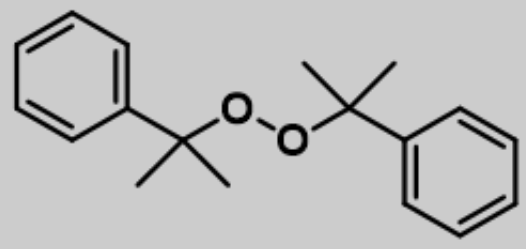
Form	70% s. in water	Liquid	80% s. in cumene	solid
Boiling point	96 ˚C	110 ˚C	153 ˚C	~400 ˚C*
Half-lifetime at 220 ˚C	~ 6 min	~ 8 s	~ 3 min	~ 2.5 s

*theoretical boiling point, as DCP decomposes beforehand

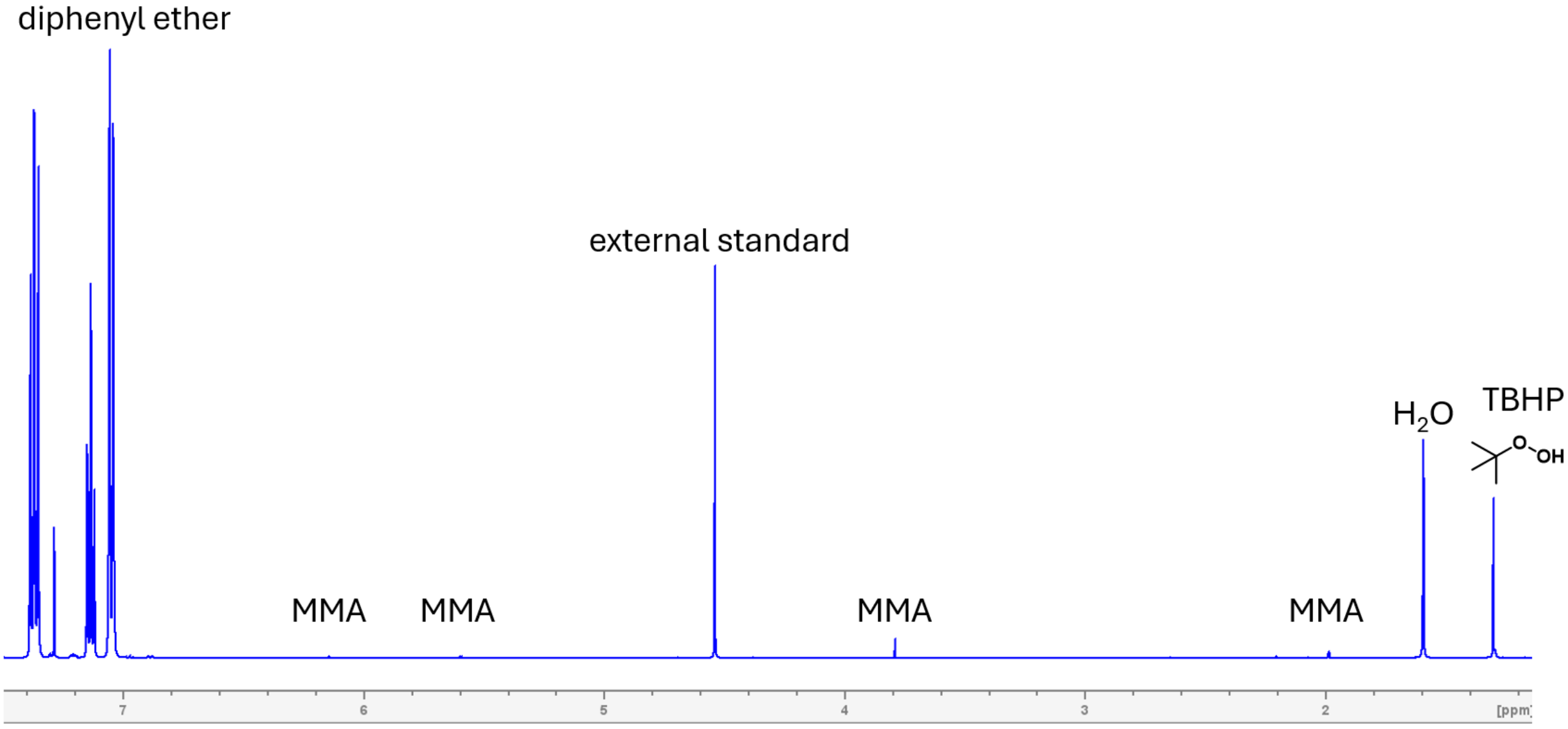

Figure S3 1H NMR spectrum of liquid collected in the receiving flask for the reaction with TBHP as a HAT agent. Presence of a high-intensity sharp singlet at 1.3 ppm confirms that TBHP was distilled from the reaction mixture.

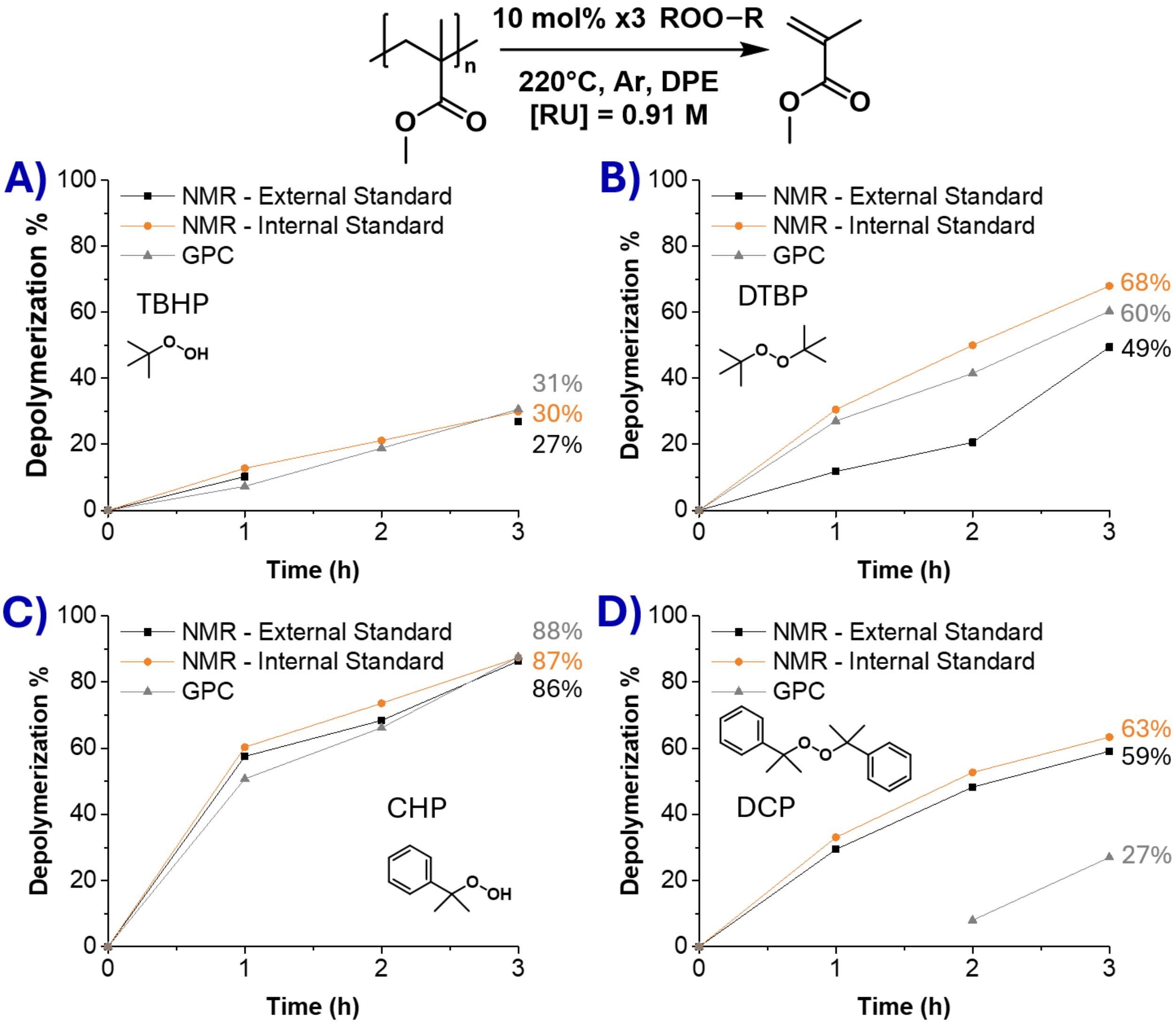

Figure S4 Results of the reagent screening depolymerization reactions - comparison of results obtained through ^{1}H NMR with external standard, internal standard, and GPC for A) TBHP; B) DTBP; C) CHP; and D) DCP. These results are for the PMMA depolymerizations at 220 °C, with three 10 mol% reagent additions respective to [RU] after each consecutive hour. [RU] = 0.91 M; 12.5 ml total reaction volume; 30 min deoxygenation prior to heating.

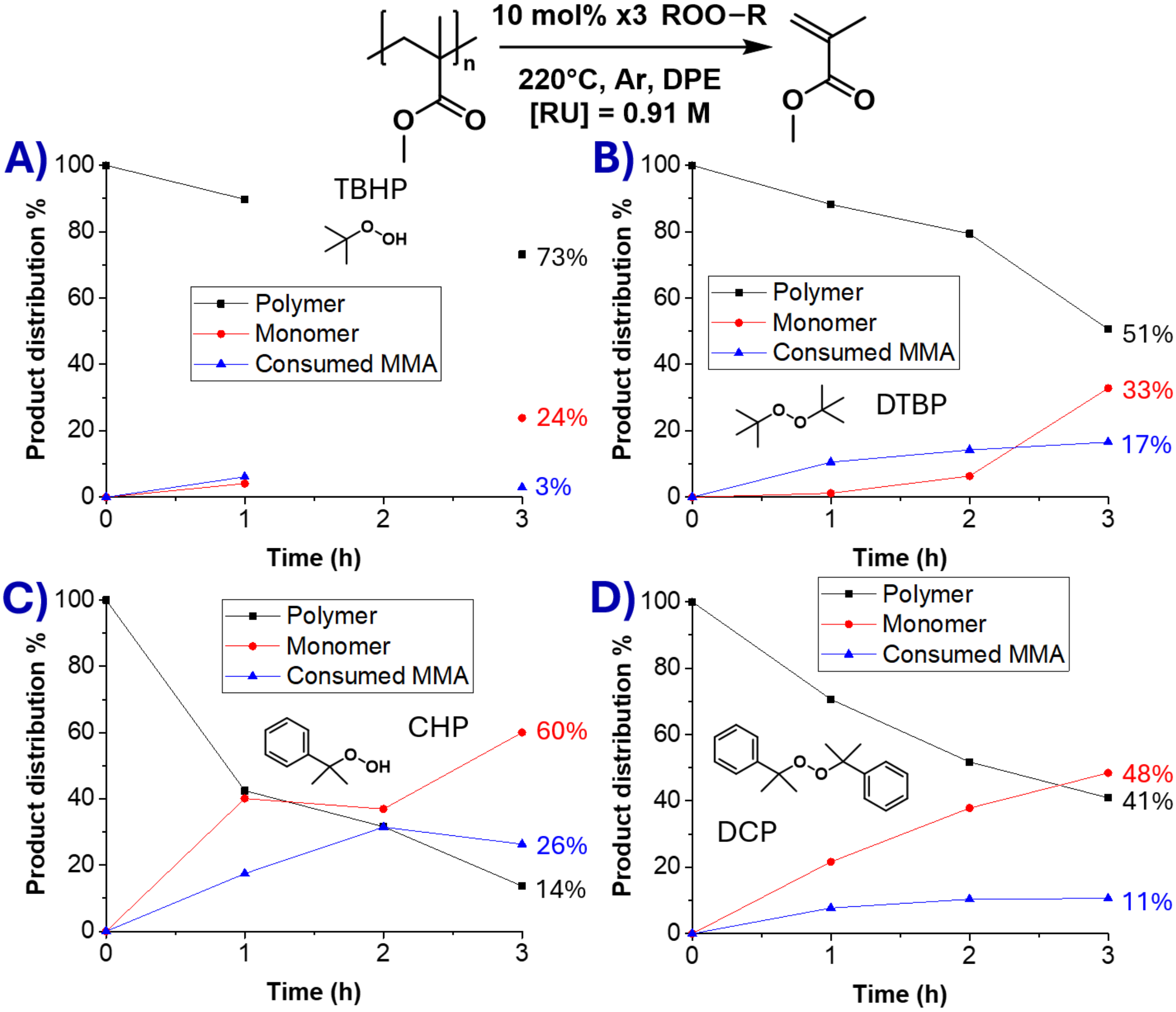

Figure S5 Results of the reagent screening depolymerization reactions - conversion of PMMA into MMA and consumed monomer, calculated using ^{1}H NMR with an external standard, for A) TBHP; B) DTBP; C) CHP; and D) DCP. These results are for the PMMA depolymerizations at 220 °C, with three 10 mol% reagent additions respective to [RU] after each consecutive hour. [RU] = 0.91 M; 12.5 ml total reaction volume; 30 min deoxygenation prior to heating.

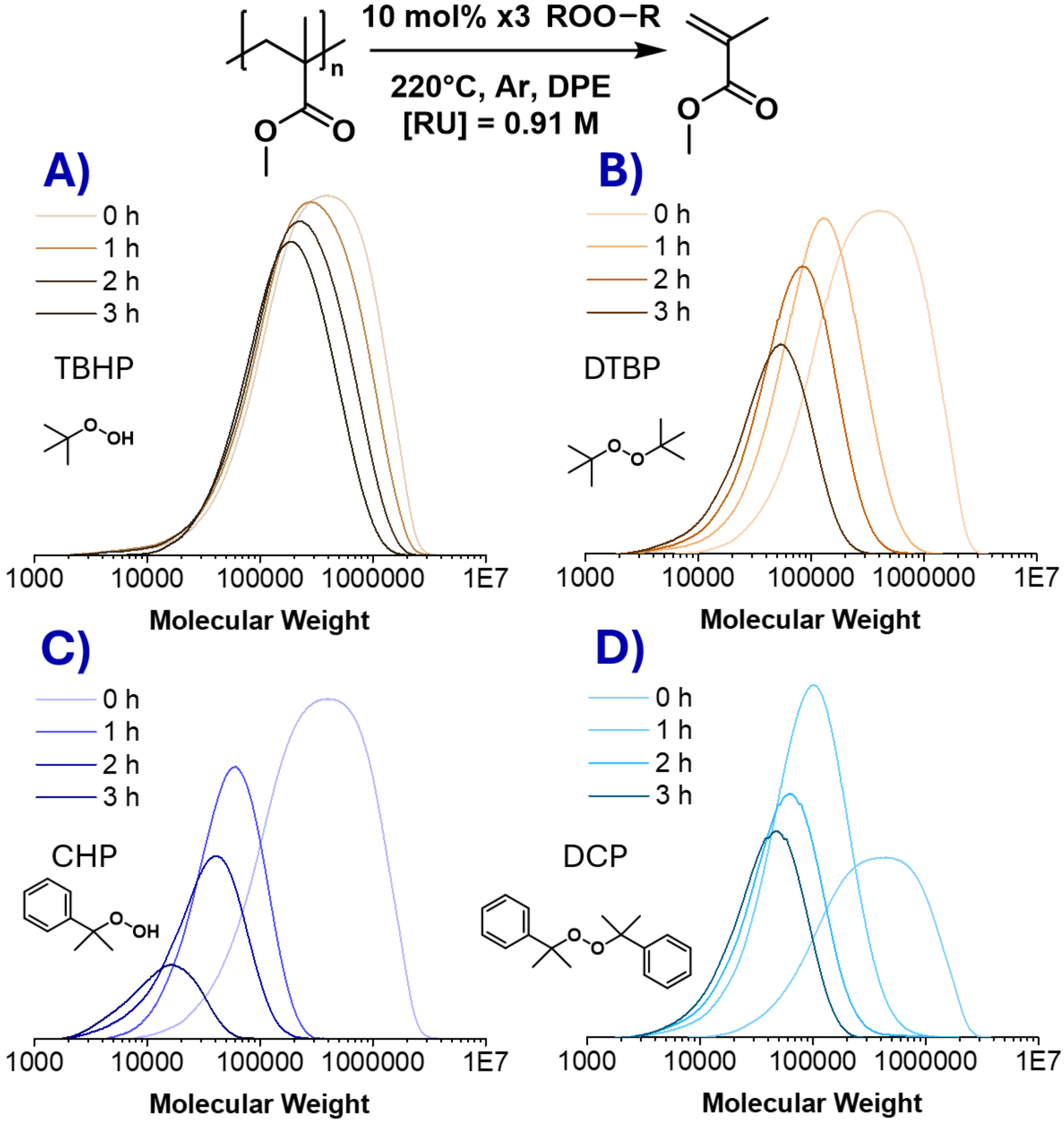

Figure S6 Results of the reagent screening depolymerization reactions - GPC traces showing depletion of area under the curve as reaction progresses for A) TBHP; B) DTBP; C) CHP; and D) DCP. These results are for the PMMA depolymerizations at 220 °C, with three 10 mol% reagent additions respective to [RU] after each consecutive hour. [RU] = 0.91 M; 12.5 ml total reaction volume; 30 min deoxygenation prior to heating.

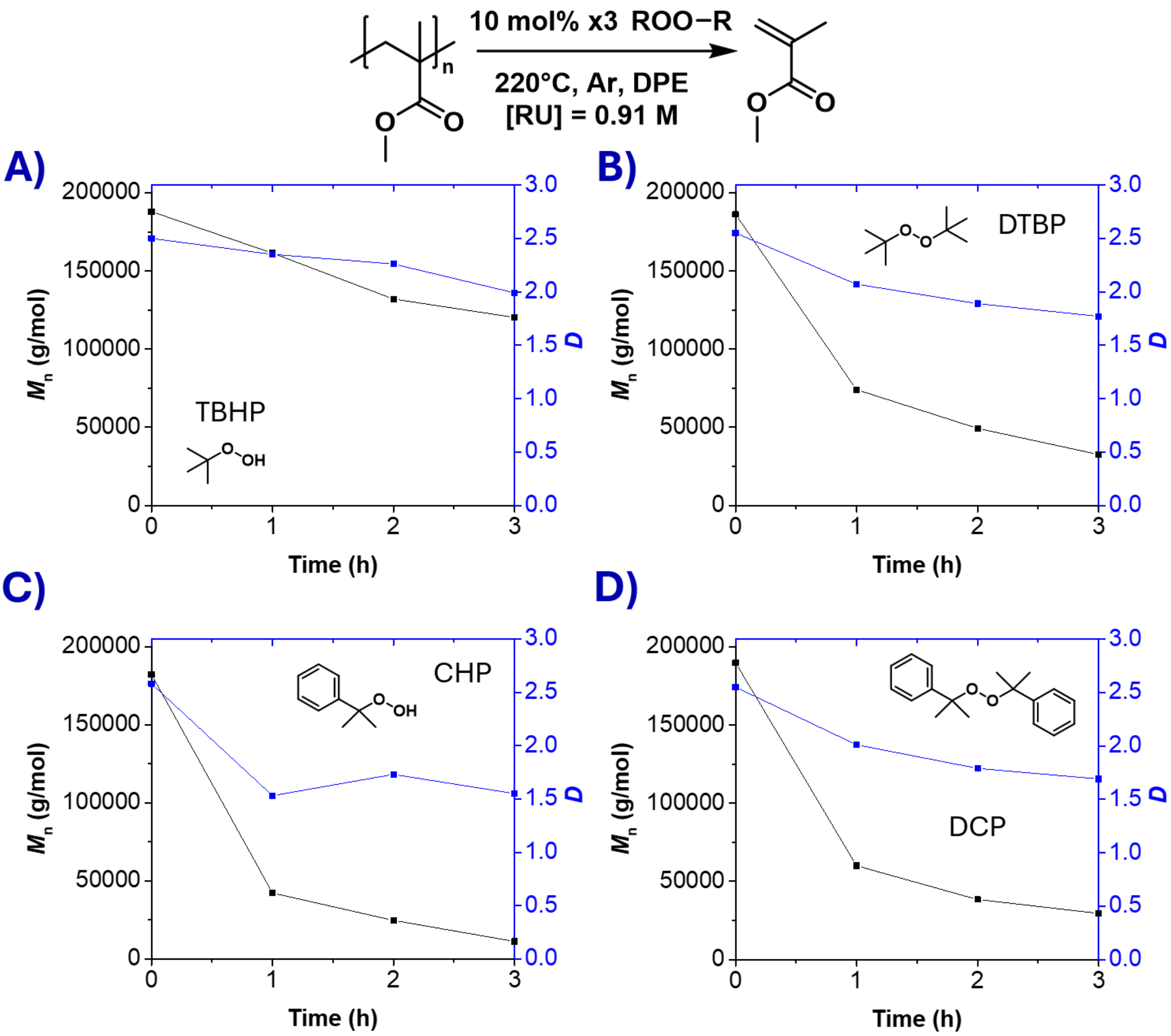

Figure S7 Results of the reagent screening depolymerization reactions - M_n and $Đ$ changes as reaction progresses for A) TBHP; B) DTBP; C) CHP; and D) DCP. These results are for the PMMA depolymerizations at 220 °C, with three 10 mol% reagent additions respective to [RU] after each consecutive hour. [RU] = 0.91 M; 12.5 ml total reaction volume; 30 min deoxygenation prior to heating.

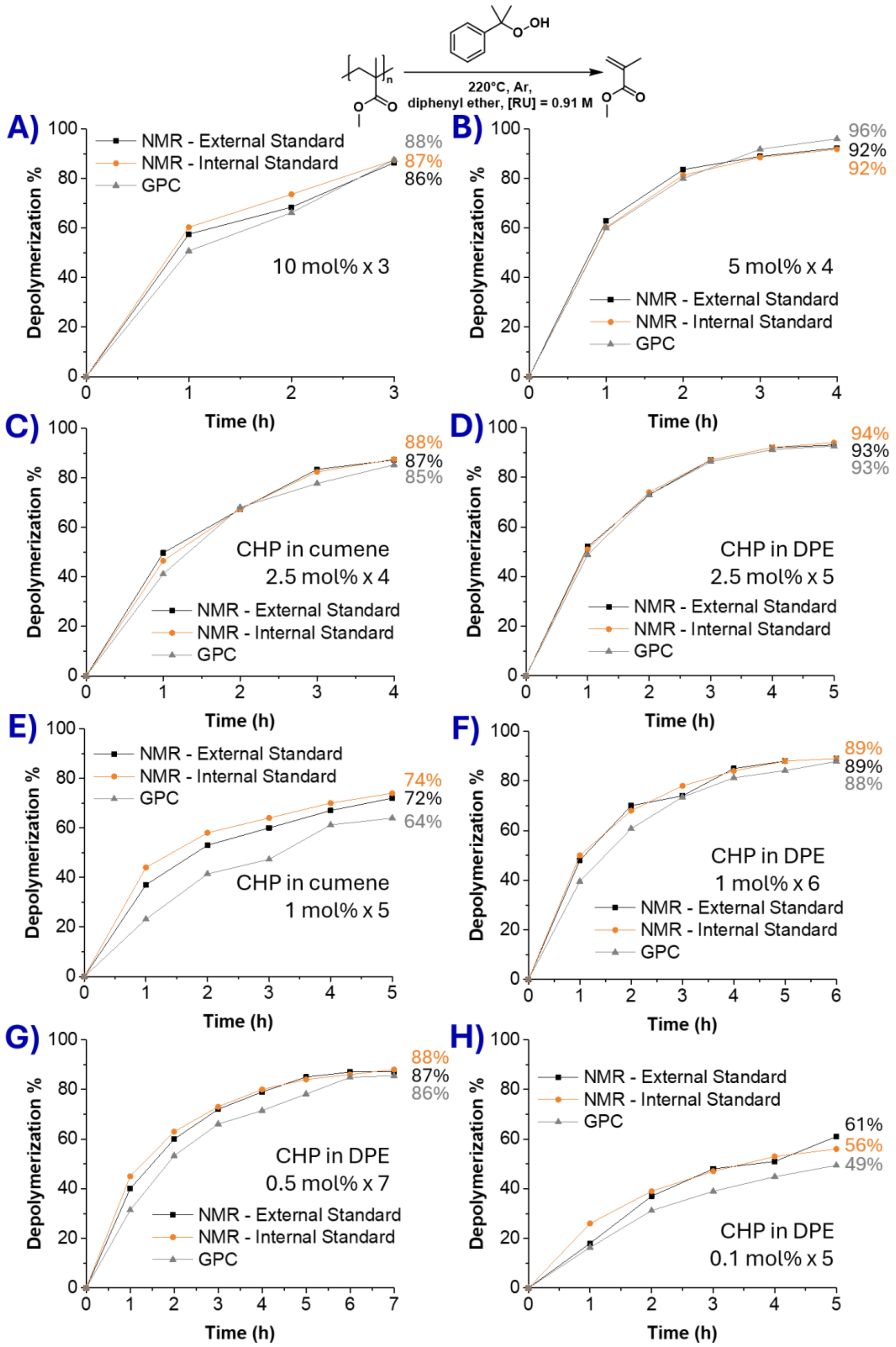

Figure S8 Results of the CHP batch-size optimization for the PMMA depolymerization at 220 °C, with semi-batch CHP additions each hour - comparison of results obtained through ^{1}H NMR with external standard, internal standard, and GPC for batch sizes relative to [RU] A) 10 mol%; B) 5 mol%; C) 2.5 mol% (CHP in cumene); D) 2.5 mol% (CHP in DPE); E) 1 mol% (CHP in cumene); F) 1 mol% (CHP in DPE); G) 0.5 mol% (CHP in DPE); and H) 0.1 mol% (CHP in DPE). The reactions were stopped when depolymerization % plateaued. [RU] = 0.91 M; 12.5 ml total reaction volume; 30 min deoxygenation prior to heating.

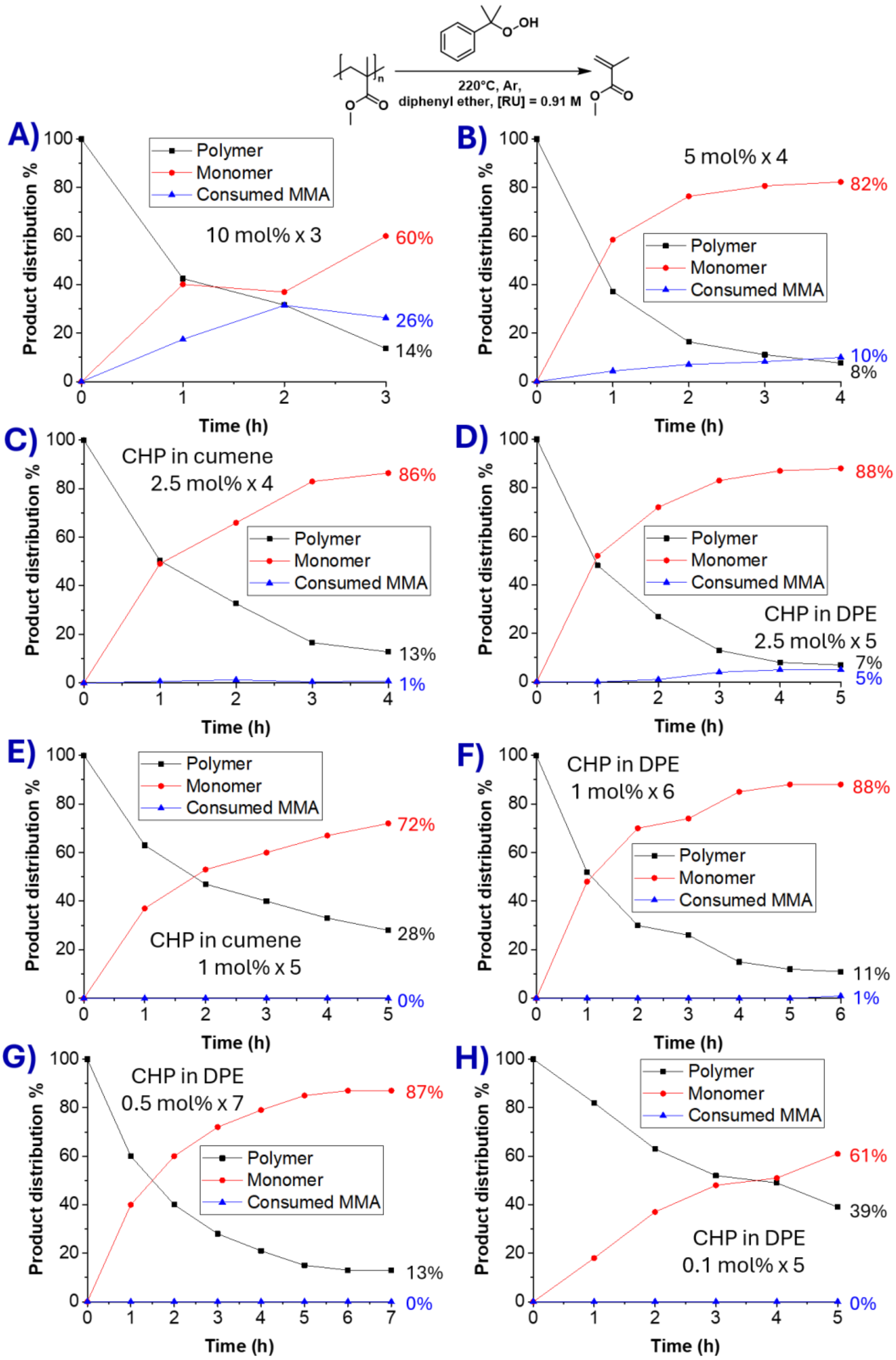

Figure S9 Results of the CHP batch-size optimization for the PMMA depolymerization at 220 °C, with semi-batch CHP additions each hour - conversion of PMMA into MMA and consumed monomer, calculated using ^{1}H NMR with an external standard, for batch sizes relative to [RU] A) 10 mol%; B) 5 mol%; C) 2.5 mol% (CHP in cumene); D) 2.5 mol% (CHP in DPE); E) 1 mol% (CHP in cumene); F) 1 mol% (CHP in DPE); G) 0.5 mol% (CHP in DPE); and H) 0.1 mol% (CHP in DPE). The reactions were stopped when depolymerization % plateaued. [RU] = 0.91 M; 12.5 ml total reaction volume; 30 min deoxygenation prior to heating.

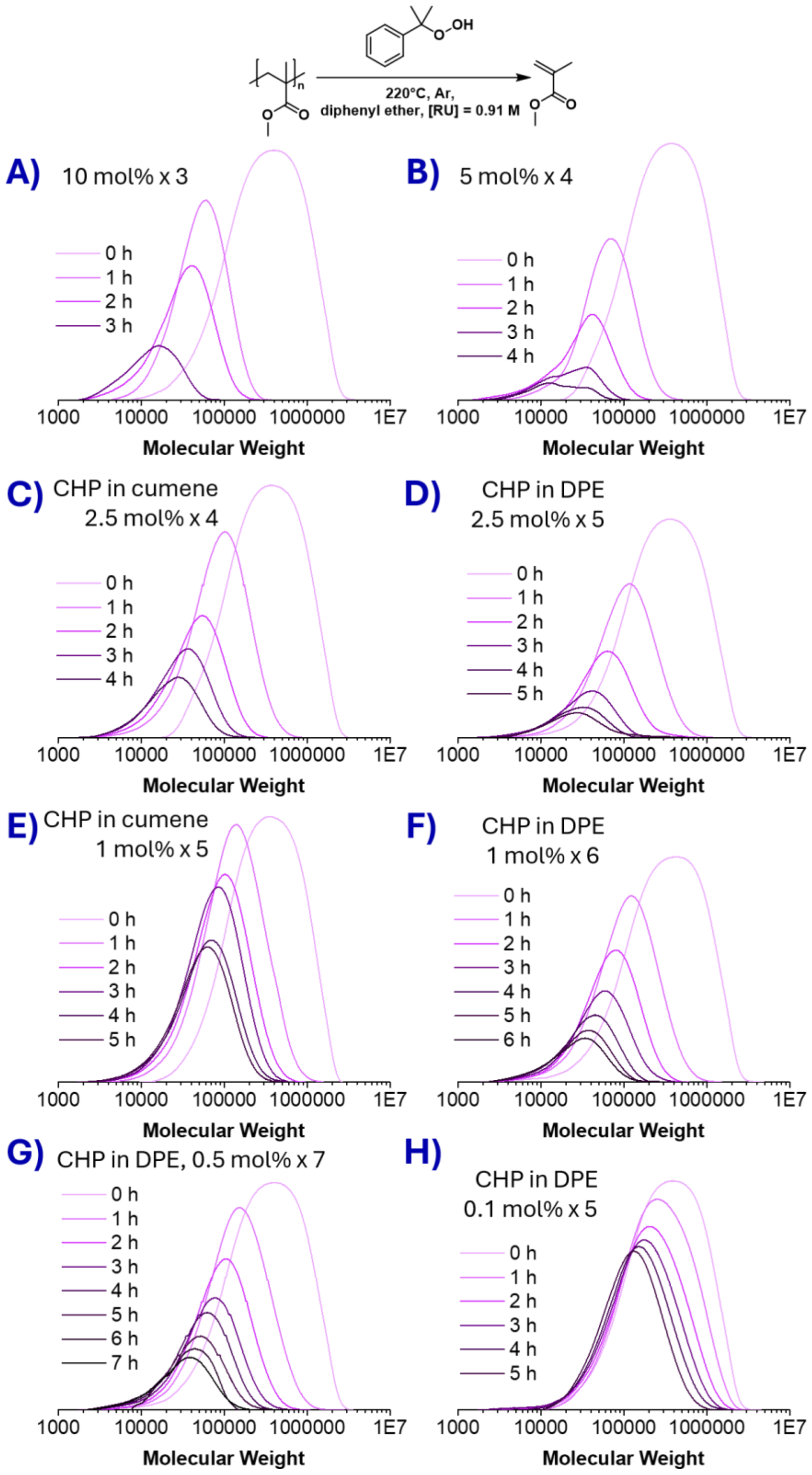

Figure S10 Results of the CHP batch-size optimization for the PMMA depolymerization at 220 °C, with semi-batch CHP additions each hour - GPC traces showing depletion of area under the curve as reaction progresses for batch sizes relative to [RU] A) 10 mol%; B) 5 mol%; C) 2.5 mol% (CHP in cumene); D) 2.5 mol% (CHP in DPE); E) 1 mol% (CHP in cumene); F) 1 mol% (CHP in DPE); G) 0.5 mol% (CHP in DPE); and H) 0.1 mol% (CHP in DPE). The reactions were stopped when depolymerization % plateaued. [RU] = 0.91 M; 12.5 ml total reaction volume; 30 min deoxygenation prior to heating.

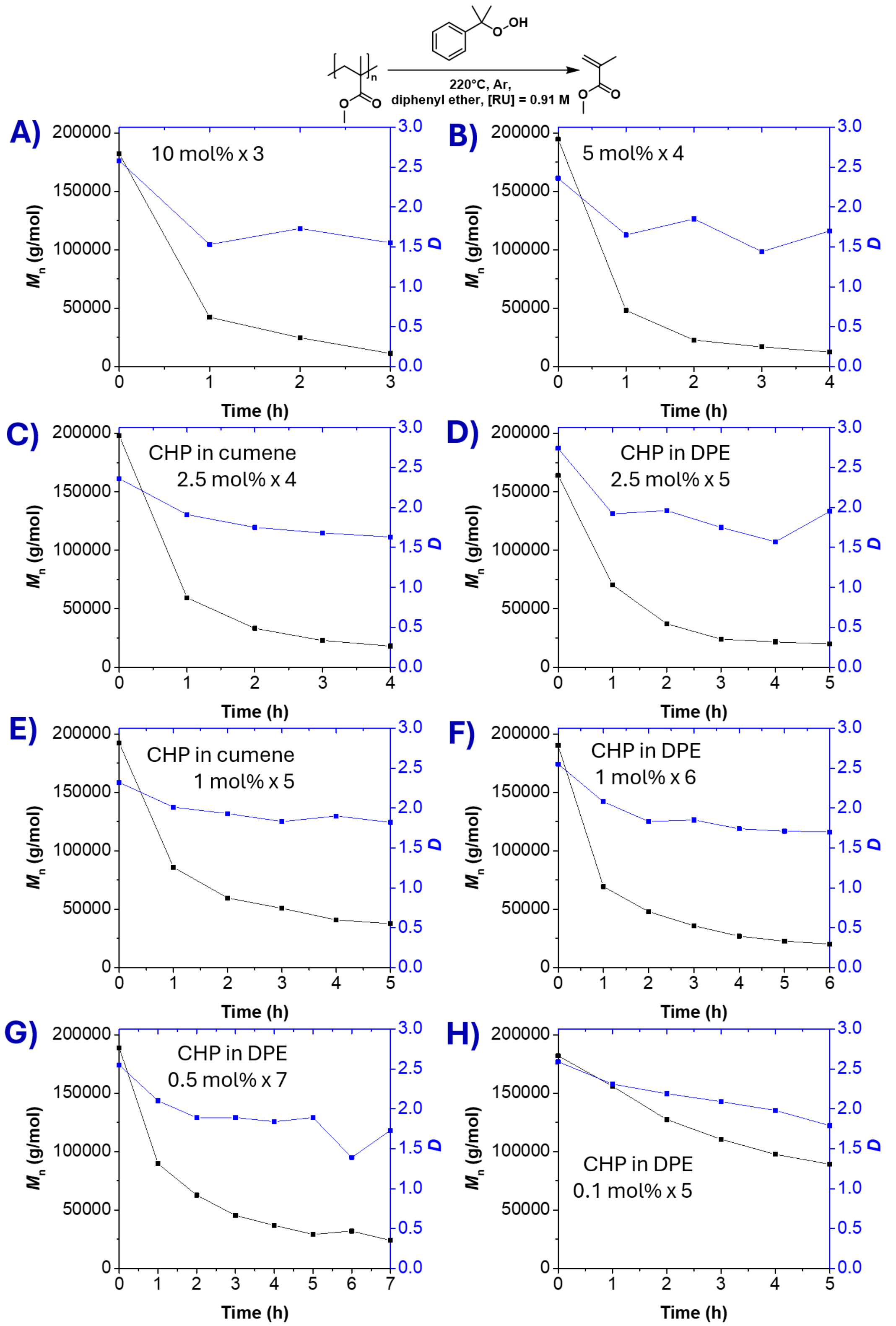

Figure S11 Results of the CHP batch-size optimization for the PMMA depolymerization at 220 °C, with semi-batch CHP additions each hour - M_n and *Đ* changes as reaction progresses for batch sizes relative to [RU] A) 10 mol%; B) 5 mol%; C) 2.5 mol% (CHP in cumene); D) 2.5 mol% (CHP in DPE); E) 1 mol% (CHP in cumene); F) 1 mol% (CHP in DPE); G) 0.5 mol% (CHP in DPE); and H) 0.1 mol% (CHP in DPE). The reactions were stopped when depolymerization % plateaued. [RU] = 0.91 M; 12.5 ml total reaction volume; 30 min deoxygenation prior to heating.

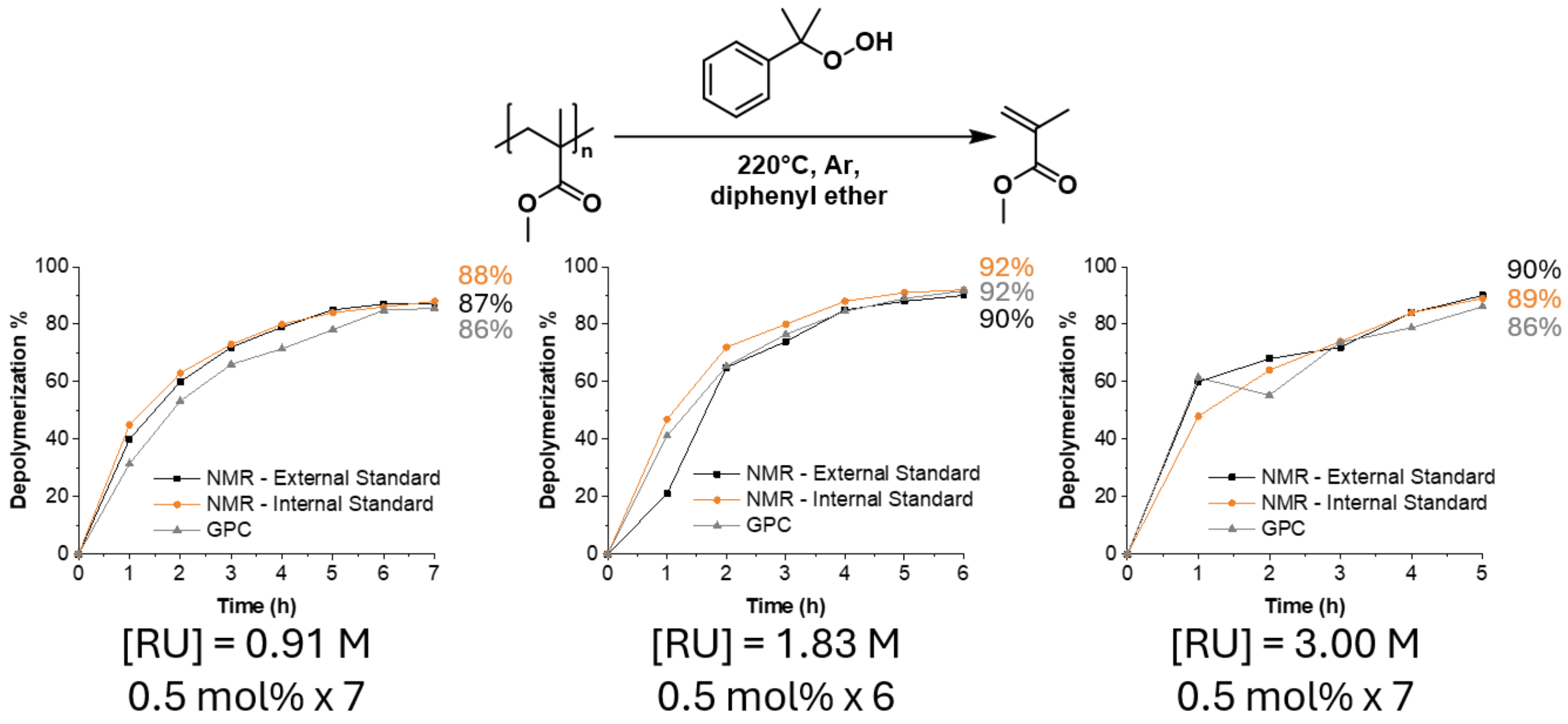

Figure S12 Results of the varied [RU] concentration depolymerization reactions - comparison of results obtained through ^{1}H NMR with external standard, internal standard, and GPC for A) [RU] = 0.91 M; B) [RU] = 1.83 M; and C) [RU] = 3.00 M. These results are for the PMMA depolymerization at 220 °C, with 0.5 mol% CHP semi-batch additions respective to [RU] each consecutive hour. 12.5 ml total reaction volume; 30 min deoxygenation prior to heating. Reaction with [RU] = 0.91 M was stopped after 7 h, [RU] = 1.83 M after 6 h, and [RU] = 3.00 M after 5 h.

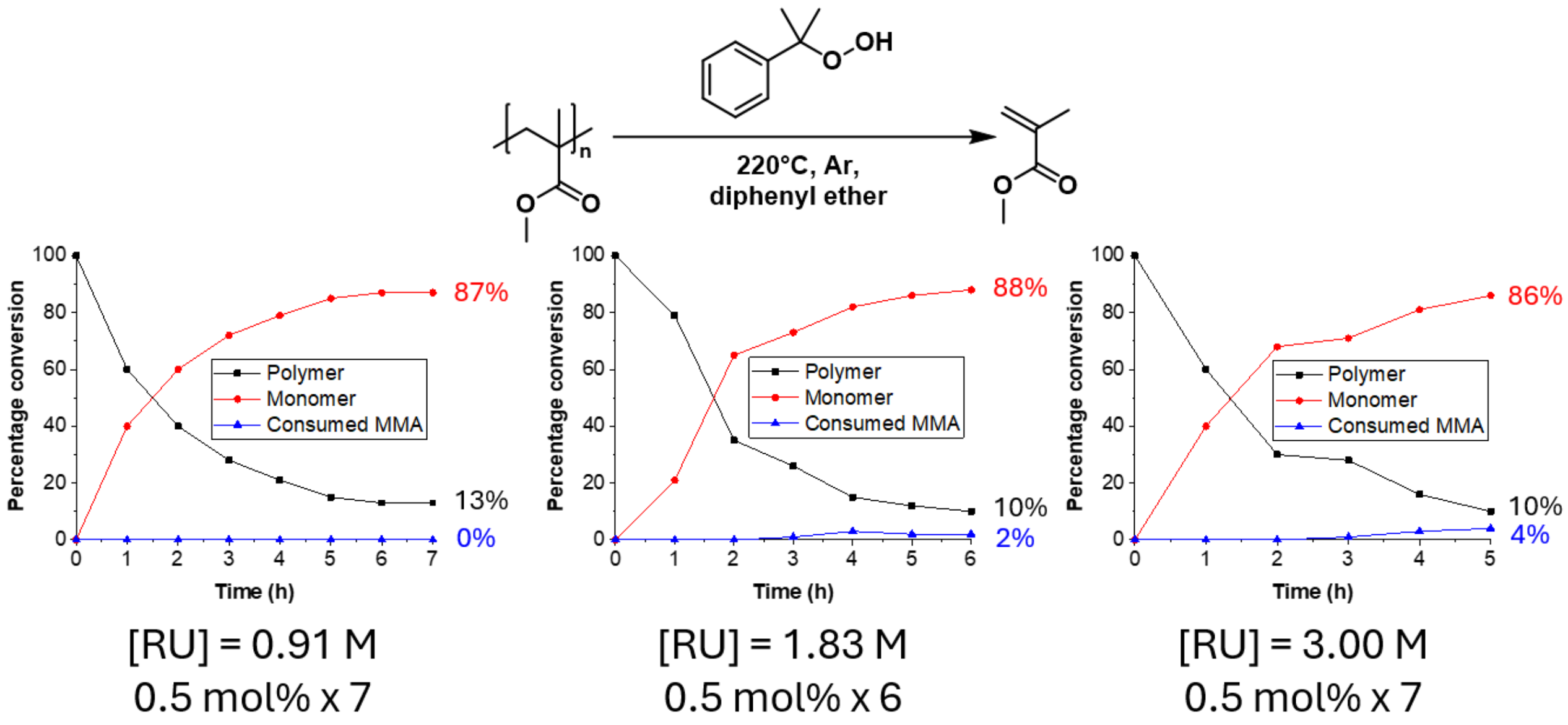

Figure S13 Results of the varied [RU] concentration depolymerization reactions - conversion of PMMA into MMA and consumed monomer, calculated using ^{1}H NMR with an external standard for A) [RU] = 0.91 M; B) [RU] = 1.83 M; and C) [RU] = 3.00 M. These results are for the PMMA depolymerization at 220 °C, with 0.5 mol% CHP semi-batch additions respective to [RU] each consecutive hour. 12.5 ml total reaction volume; 30 min deoxygenation prior to heating. Reaction with [RU] = 0.91 M was stopped after 7 h, [RU] = 1.83 M after 6 h, and [RU] = 3.00 M after 5 h.

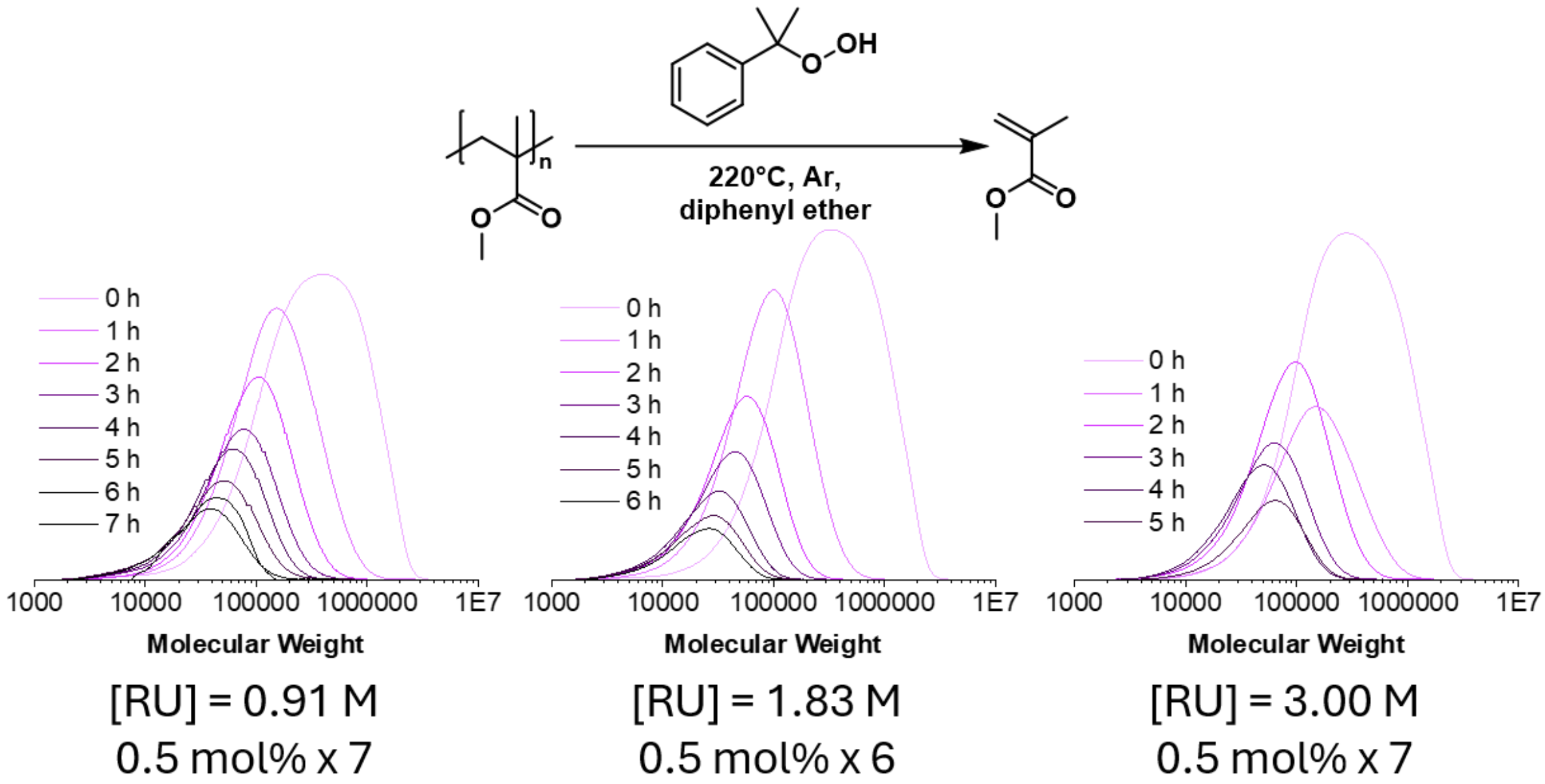

Figure S14 Results of the varied [RU] concentration depolymerization reactions - GPC traces showing depletion of area under the curve as reaction progresses for A) [RU] = 0.91 M; B) [RU] = 1.83 M; and C) [RU] = 3.00 M. These results are for the PMMA depolymerization at 220 °C, with 0.5 mol% CHP semi-batch additions respective to [RU] each consecutive hour. 12.5 ml total reaction volume; 30 min deoxygenation prior to heating. Reaction with [RU] = 0.91 M was stopped after 7 h, [RU] = 1.83 M after 6 h, and [RU] = 3.00 M after 5 h.

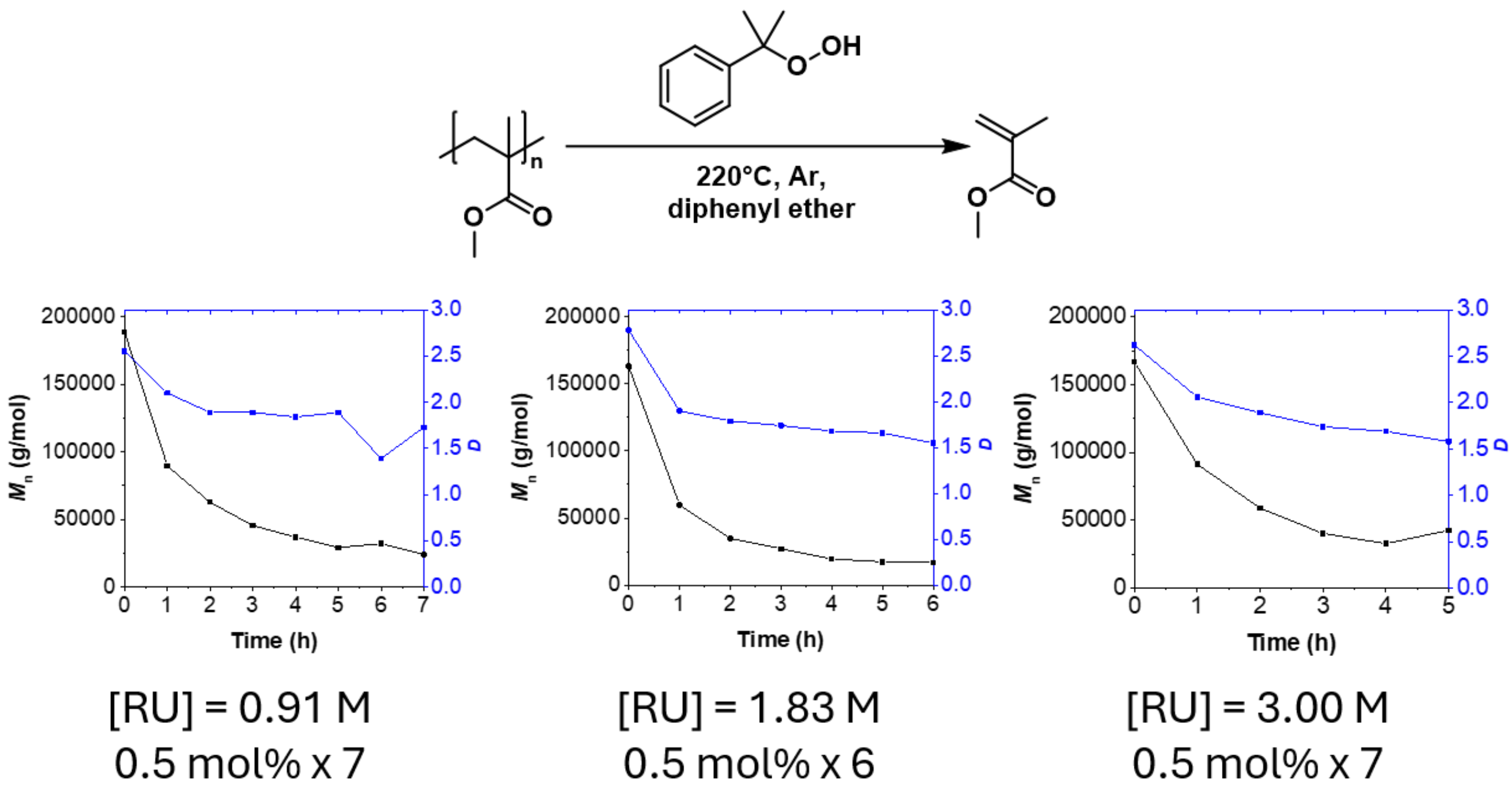

Figure S15 Results of the varied [RU] concentration depolymerization reactions - M_n and $Đ$ changes as reaction progresses for A) [RU] = 0.91 M; B) [RU] = 1.83 M; and C) [RU] = 3.00 M. These results are for the PMMA depolymerization at 220 °C, with 0.5 mol% CHP semi-batch additions respective to [RU] each consecutive hour. 12.5 ml total reaction volume; 30 min deoxygenation prior to heating. Reaction with [RU] = 0.91 M was stopped after 7 h, [RU] = 1.83 M after 6 h, and [RU] = 3.00 M after 5 h.

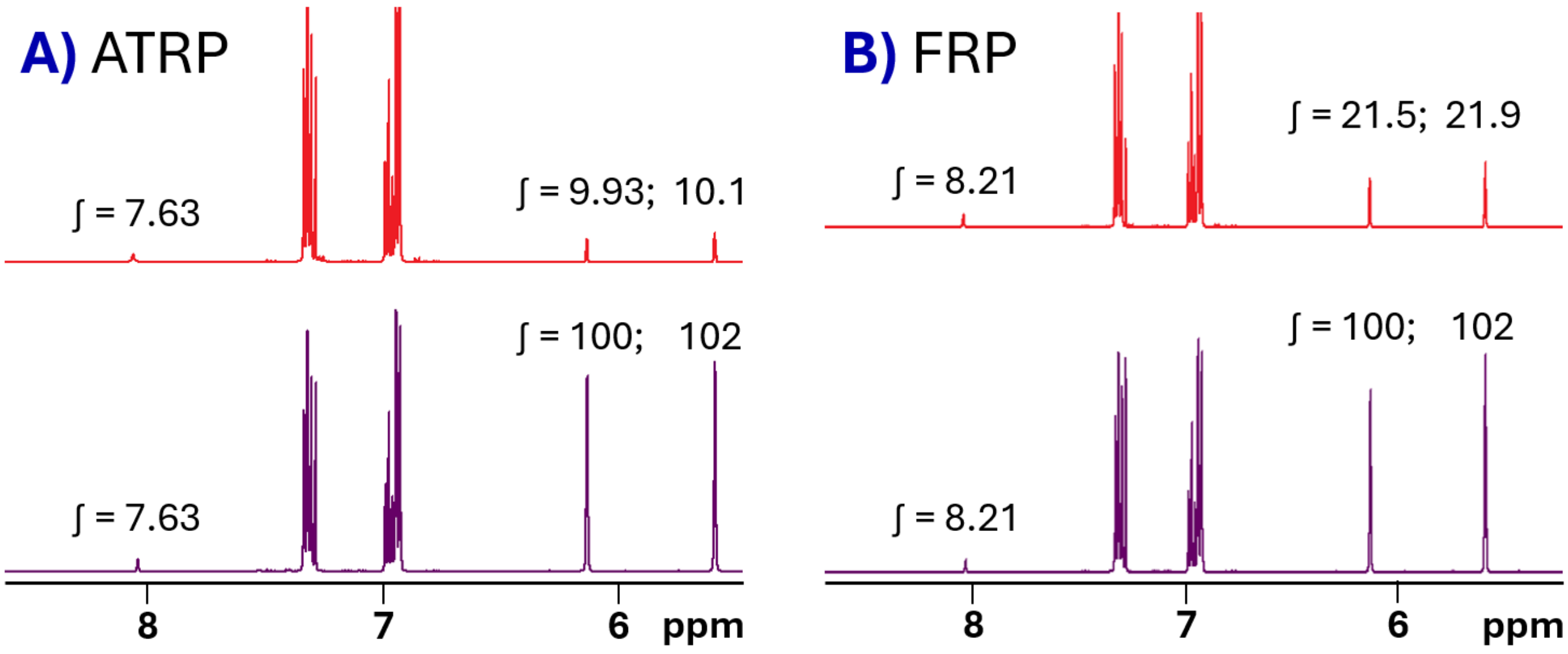

Figure S16 Results of the polymerization reactions. 1H NMR spectra showing conversion calculations for A) ATRP; and B) FRP. ARGET ATRP: 70 °C, 2 mL total reaction volume with 47% v/v anisole. Ethyl-**α**-chlorophenyl acetate : MMA : $CuCl_2$: PMDETA = 1 : 200 : 0.1 : 0.175. One drop of tin(II) 2-ethylhexanoate was added as a reducing agent. FRP: 70 °C, 2 mL total reaction volume with 48% v/v anisole. AIBN : MMA = 1 : 200. Both polymerizations were preceded by 20 min of degassing.

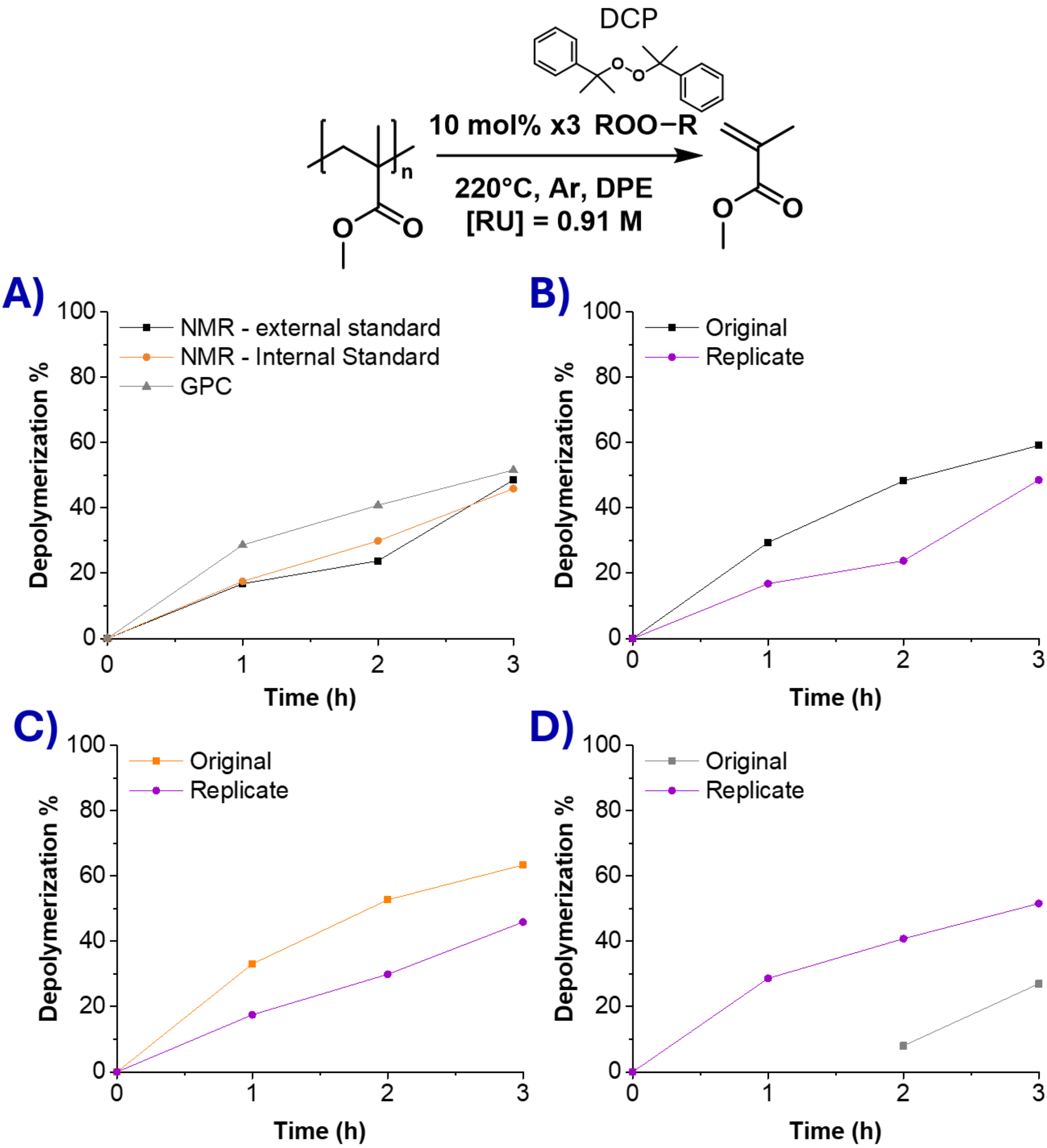

Figure S17 Results of the repeated depolymerization reaction with DCP. A) Comparison of results obtained through ^1H NMR with external standard, internal standard, and GPC. Comparison of original and repetition result from B) ^1H NMR with external standard; C) ^1H NMR with internal standard; and D) GPC. These results are for the PMMA depolymerizations at 220 °C, with three 10 mol% reagent additions respective to [RU] after each consecutive hour. [RU] = 0.91 M; 12.5 ml total reaction volume; 30 min deoxygenation prior to heating.

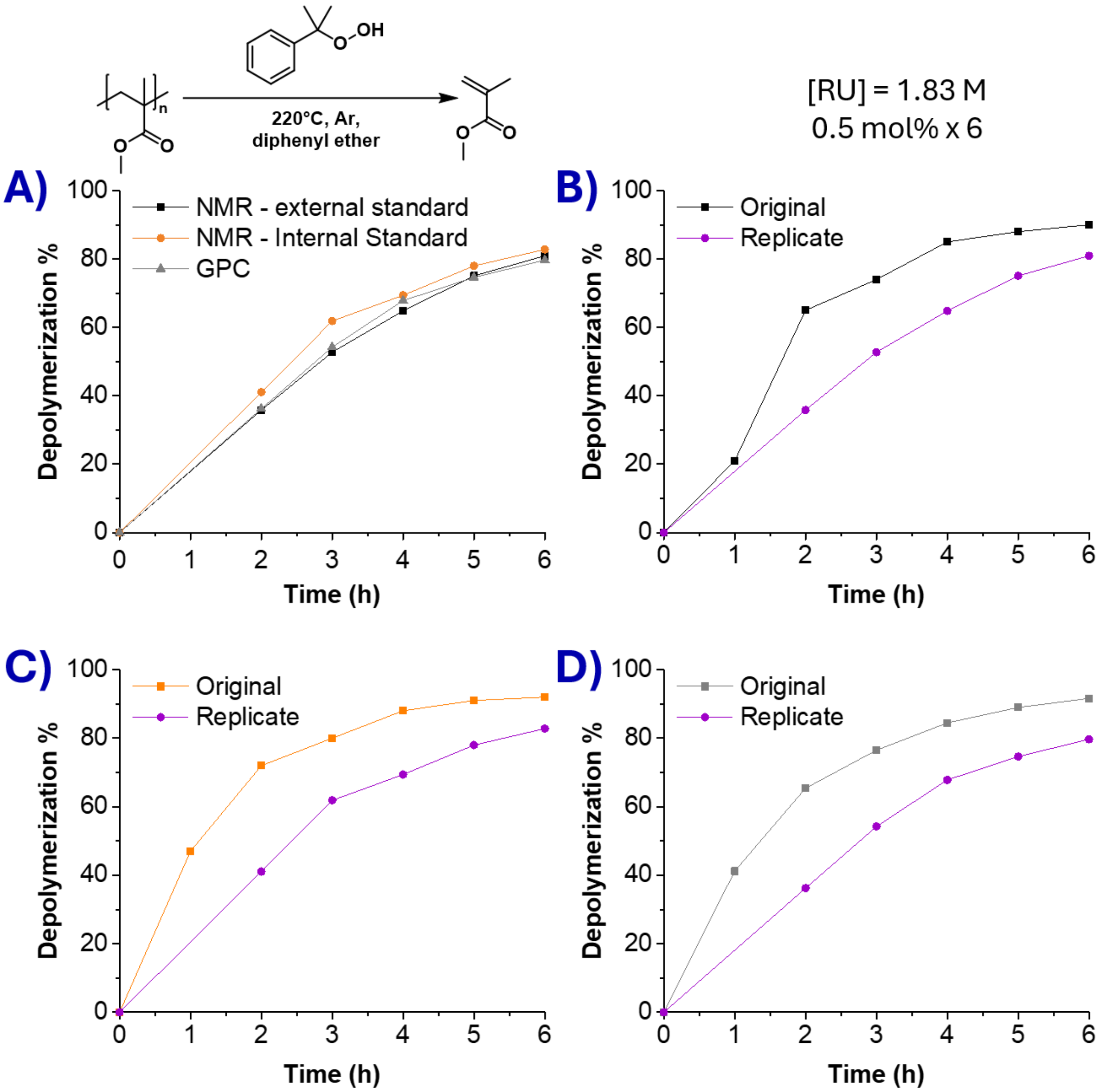

Figure S18 Results of the repeated depolymerization reaction with [RU] = 1.83 M. A) Comparison of results obtained through ^{1}H NMR with external standard, internal standard, and GPC. Comparison of original and repetition result from B) ^{1}H NMR with external standard; C) ^{1}H NMR with internal standard; and D) GPC. These results are for the PMMA depolymerizations at 220 °C, with six 0.5 mol% reagent additions respective to [RU] after each consecutive hour. [RU] = 1.83 M; 12.5 ml total reaction volume; 30 min deoxygenation prior to heating.

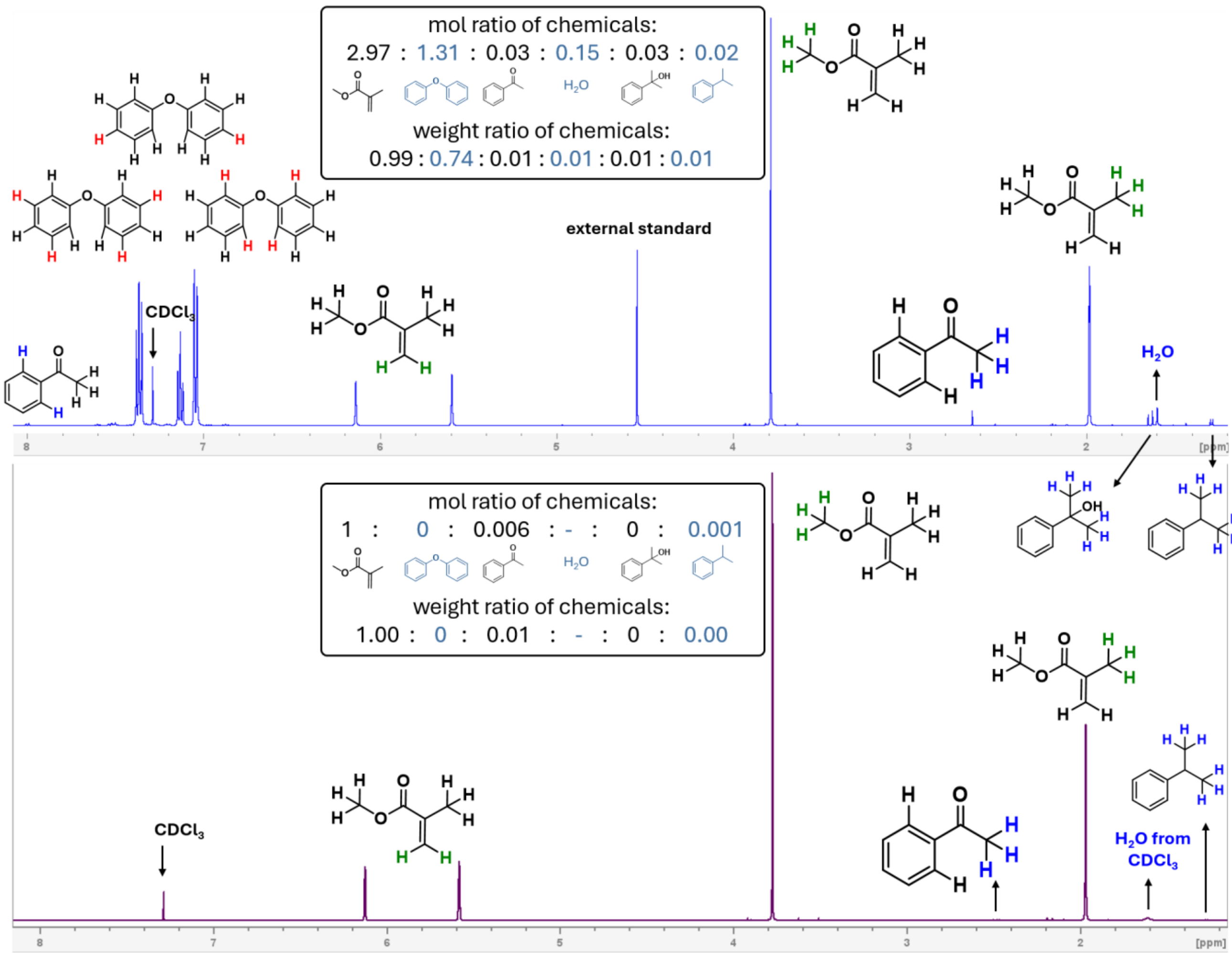

Figure S19 Comparison of crude product of depolymerization reaction with purified product. These results are for the PMMA depolymerization at 220 °C, with six 0.5 mol% reagent additions respective to [RU] after each consecutive hour. [RU] = 1.83 M; 12.5 ml total reaction volume; 30 min deoxygenation prior to heating, followed by product purification by distillation.

References

(1) Young, J. B.; Bowman, J. I.; Eades, C. B.; Wong, A. J.; Sumerlin, B. S. Photoassisted Radical Depolymerization. *ACS Macro Lett.* **2022**, *11* (12), 1390–1395. https://doi.org/10.1021/acsmacrolett.2c00603

(2) De Luca Bossa, F.; Szewczyk-Łagodzińska, M.; Magro, A.; Coskun, H. I.; Yilmaz, G.; Lin, T.-C.; DiFiglia, E.; Matyjaszewski, K. Depolymerization of Commercial Polymethacrylates Triggered by Hydroperoxides. *J. Am. Chem. Soc.* **2026**, *148* (31), 33622–33630. https://doi.org/10.1021/jacs.6c10099
